# Integrodifference models for persistence in temporally varying river environments

**DOI:** 10.1007/s00285-014-0774-y

**Published:** 2014-03-14

**Authors:** Jon Jacobsen, Yu Jin, Mark A. Lewis

**Affiliations:** 1 Department of Mathematics, Harvey Mudd College, Claremont, CA 91711 USA; 2 Department of Mathematics, University of Nebraska-Lincoln, Lincoln, NE 68588 USA; 3 Departments of Mathematical and Statistical Sciences and of Biological Sciences, University of Alberta, Edmonton, AB T6G 2G1 USA

**Keywords:** Integrodifference, Time-varying environment, Random advection kernels, Persistence, 45C05, 45R05, 92B05

## Abstract

To fully understand population persistence in river ecosystems, it is necessary to consider the effect of the water flow, which varies tremendously with seasonal fluctuations of water runoff and snow melt. In this paper, we study integrodifference models for growth and dispersal in the presence of advective flow with both periodic (alternating) and random kernel parameters. For the alternating kernel model, we obtain the principal eigenvalue of the linearization operator to determine population persistence and derive a boundary value problem to calculate it. For the random model, we establish two persistence metrics: a generalized spectral radius and the asymptotic growth rate, which are mathematically equivalent but can be understood differently, to determine population persistence or extinction. The theoretical framework and methods for calculations are provided, and the framework is applied to calculating persistence in highly variable river environments.

## Introduction

### Background

Stream and river ecosystems are shaped by their physical environment of unidirectional water flow. Questions of population persistence in river ecosystems must necessarily consider the effect of the water flow on populations in space and time. To complicate matters, this water flow can vary tremendously with seasonal fluctuations of water runoff and snow melt.

How can populations persist in streams when they are being constantly washed downstream? This so-called *drift paradox* (Muller 1982) has engaged biologists and mathematicians in a series of modeling efforts using reaction–advection–diffusion equations to describe the population densities in space and time. The early paper of Speirs and Gurney (2001) uses a modification of Fisher’s equation that includes advection to show the existence of a critical flow rate in the stream, below which the populations will persist, and above which the population will wash out, much as a chemostat population will persist or wash out in low flow and high flow conditions.

The approach of Speirs and Gurney (2001) employs classical mathematical methods of population spreading speeds and critical domain size. The spreading speed for Fisher’s equation, $2\sqrt{r\!D}$ where $r$ is the intrinsic growth rate and $D$ is the diffusion coefficient (Aronson and Weinberger 1975), yields the critical advection velocity $v_c$, below which stream populations will persist, and above which they will wash out. This can be understood intuitively: when the advection velocity $v$ exactly matches the spreading speed $2\sqrt{r\!D}$, the population is washed downstream by water flow at the same speed it is moving upstream by the combined effects of growth ($r$) and diffusion ($D$). Speirs and Gurney (2001) show the critical domain size $L_c$ exists for all advection velocities that lie below the critical value $(0<v<v_c)$ and that the critical domain size approaches infinity as $v$ approaches $v_c$. Biologically, this is interpreted as implying that stream populations will persist if the advection speed falls below a threshold value, and there is a sufficiently large stretch of stream available. This theory has been tested empirically by Walks (2007) who related the persistence of plankton in flowing water to stream advection velocities. A mathematical review of the ideas in Speirs and Gurney (2001) can be found in Lewis et al. (2009).

Extensions to the theory have focused on increasingly realistic models for the stream populations. These include stationary and mobile compartments to describe subpopulations on the benthos and in the stream (Pachepsky et al. 2005), non-diffusive dispersal of stream populations that can include long-distance jumps (Lutscher et al. 2005), spatially varying stream environments (Lutscher et al. 2006), spatial interactions between competitors in the stream environment (Lutscher et al. 2007) and periodic fluctuations in environmental conditions (Jin and Lewis 2011, 2012).

Despite these extensions to the theory, the models have been limited to the case where the stream environment is predictable. Although convenient from a modelling perspective, this is inaccurate. For example stream flows not only vary by an order of magnitude between spring and fall seasons (Abrahamsson and Hakanson 1998), they also vary unpredictably from year to year (Anderson et al. 2006). While some models exist for spreading populations in randomly fluctuating (Neubert et al. 2000) environments, none have investigated persistence and spread in environments such as streams, where unidirectional flow predominates.

In this paper we investigate persistence of populations in periodic and randomly fluctuating environments with predominantly unidirectional flow. Our mathematical model is based on a discrete-time and continuous-space dynamical system that takes the form of an integrodifference equation. In the next section we develop a modelling background for integrodifference models.

### Integrodifference models

We consider organisms with separate growth and dispersal stages. Dispersal is assumed to be continuous in space and occurring over a fixed time interval (the *dispersal event*) while growth is independent of space, but depends on the local population density. Denoting $n_t(x)$ as the population density at stage $t$, the growth dynamics are modeled by

1 $$\begin{aligned} f(n_t )=n_t(x)g(n_t(x)), \end{aligned}$$

where $f$ is a nonnegative monotonically increasing function. The function $g(n)$ is the per capita growth rate and we assume the maximum per capita growth rate is found as $n$ approaches zero. The dispersal dynamics are modelled by the integral equation

2 $$\begin{aligned} I[n_t](x) = \int _{\varOmega } K(x,y) \, n_{t}(y) \, dy, \end{aligned}$$

where the dispersal kernel $K(x,y)$ models the probability density associated with an individual, that starts at $y$, settling at $x$ during the dispersal event. We assume that $K$ is a continuous nonnegative function with area one when integrated with respect to $x$ over the real line for all fixed y. The combined model for growth and dispersal is then given by the the nonlinear integrodifference model

3 $$\begin{aligned} n_{t+1} (x) = \int _{\varOmega } K(x,y) \, f(n_t(y)) \, dy. \end{aligned}$$

Equations of the form (3) were first formulated to study gene flow and selection (Slatkin 1973), and were only later applied in ecological settings (Kot and Schaffer 1986). As written, Eq. (3) assumes an unstructured population that grows in the stream benthos, and disperses through the stream and settles back to the benthos each time step. Although the population is assumed to be unstructured, an extension of the model can be used to describe stage-structured populations where dispersal varies from stage to stage (Lutscher and Lewis 2004).

We consider a habitat $\varOmega = [x_0,y_0]$ for some $x_0 < y_0$. For such a bounded domain $\varOmega$ the model (3) assumes that the organism can disperse across the boundary, but there is no source term from outside the boundary. This is the case if conditions outside $\varOmega$ are unfavorable to growth and survival or if the organism cannot disperse back into the habitat $\varOmega$ once it has left. This would be the case for a stretch of suitable habitat in a stream, surrounded by unsuitable habitat, where the organism cannot survive. A non-aquatic example is of a plant whose seeds are blown across the edge of a field into a parking lot or other unsuitable region.

#### The dispersal kernel

**Fig. 1:**
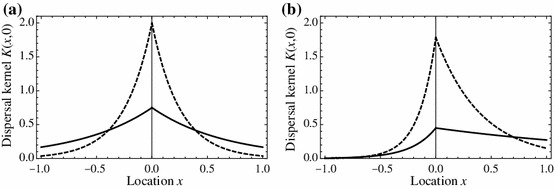
Sample dispersal kernels: **a** Laplace kernel (4) with $a=4$ (*dashed*) and $a=1.5$ (*solid*); **b** asymmetric Laplace kernel (5) with identical parameter values as **a** and $v=4$

The dispersal kernel $K$ can take a variety of forms. If an individual at $y$ moves randomly for a fixed amount of time $T$ and then settles, the dispersal kernel is a Gaussian with variance $2DT$ where $D$ is the diffusion coefficient associated with the random movement. Alternatively, if the randomly moving individual settles at a constant rate $\beta > 0$ then, after a sufficiently long period of time, the kernel approaches a Laplace distribution

4 $$\begin{aligned} K(x,y) = \frac{a}{2} e^{-a|x-y|} \end{aligned}$$

where $a = \sqrt{\frac{\beta }{D}}$ (Neubert et al. 1995). Figure 1a shows two sample Laplace kernels with $a=4$ and $a=1.5$ for the habitat $\varOmega = [-1,1]$. Note that the kernel for the larger value $a=4$ corresponds to a higher probability of the organism settling in $\varOmega$, which is consistent with the higher value of $a$ arising from a larger settling rate or a smaller diffusion coefficient.

If, in addition, an organism experiences a unidirectional flow with velocity $v$ (e.g., stream flow or wind) the kernel takes the form

5 $$\begin{aligned} K(x,y) = {\left\{ \begin{array}{ll} A \, e^{a_1 (x-y)} &{}\quad x < y \\ A \, e^{a_2 (x-y)} &{}\quad x \ge y, \end{array}\right. } \end{aligned}$$

where the rate constants $a_{i}$ are defined in terms of the advection velocity $v$, settling rate $\beta$, and diffusion coefficient $D$ by

$$\begin{aligned} a_{1,2} = \frac{v}{2D} \pm \sqrt{\frac{v^2}{4D^2} + \frac{\beta }{D}} \qquad \text { and } \qquad A = \frac{a_1 a_2}{a_2 - a_1} = \frac{\beta }{\sqrt{v^2 + 4 \beta D}} \end{aligned}$$

(Lutscher et al. 2005). Figure 1b shows the same kernels in Fig. 1a, but now with an advective velocity $v=4$. The derivation of (5) assumes a separation of time scales between settling and dispersal to employ a partial differential equation describing the time-dependent probability density $z(t,x)$ of an individual that starts moving at point $y$ at time $t=0$ via advection (due to the flow), diffusion (as a first approximation to variability in the flow speed and direction), and settles at rate $\beta$:

6 $$\begin{aligned} z_{t}=Dz_{xx}-vz_x-\beta z,\qquad z(0,x)=\delta _0(x-y). \end{aligned}$$

The density of settling points at $x$ is then given by

7 $$\begin{aligned} k(x)=\int _0^\infty \beta z(t,x)\,dt. \end{aligned}$$

Integrating (6) over $0<t<\infty$ yields an ordinary differential equation for $k$

8 $$\begin{aligned} \frac{D}{\beta } \, k''(x) - \frac{v}{\beta } k'(x) - k(x) = -\delta _0(x-y) \end{aligned}$$

defined on the real line $-\infty <x<\infty$ whose solution is $K(x,y)$, as defined by (5). Here it is assumed that the dispersing individual does not modify its movement in response to the domain boundary. If such behaviour is included, it gives rise to boundary conditions for (8) which modifies the associated Green’s function for $K$ (Lutscher et al. 2005, Appendix E).

Two quantities that are derived from the dispersal kernel and are relevant to our modelling considerations are the *dispersal success function* and the *redistribution function*. The dispersal success function $s(y)$ indicates the probability that an individual starting at $y$ successfully settles in the habitat $\varOmega$ after the dispersal event:

9 $$\begin{aligned} s(y) = \int _{\varOmega } K(x,y) \, d x. \end{aligned}$$

Since $K(x,y) \ge 0$ and $\int _{\mathbb {R}} K(x,y_0) \, dx = 1$ for any $y_0$ we have $0 \le s(y) \le 1$. The redistribution function $r(x)$, on the other hand, corresponds to an area release experiment; if $N$ individuals are released uniformly over the patch, then after one dispersal event the expected density of individuals is $N r(x)$ where

10 $$\begin{aligned} r(x) = \int _{\varOmega } K(x,y) \, d y. \end{aligned}$$

**Fig. 2:**
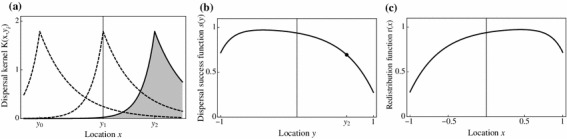
Asymmetric Laplace kernel (**a**) and the corresponding dispersal success function (**b**) and redistribution function (**c**). Parameter values for the kernel are $a=4$ and $v=4$ in Eq. (5). The *dot* in **b** indicates the dispersal success for location $y_2$ which corresponds to the area of the *shaded* kernel in **a**

By way of contrast with the dispersal success function $s$, the redistribution function $r$ needs not be bounded above by $1$ for all $x$. However, for symmetric kernels the two functions are identical. Figure 2 shows an example of the dispersal success and redistribution functions for an asymmetric Laplace kernel on $\varOmega =[-1,1]$.

The dispersal success function provides a means to approximate the principal eigenvalue of the linearization of (3), which itself can be used as a measure of population persistence (see Sect. 1.3.1). More specifically, let $\lambda _1(K)$ denote the principal eigenvalue of the linearization of (3) at the equilibrium solution $n_*(x) = 0$ and $\phi$ an associated positive eigenfunction, i.e.,

11 $$\begin{aligned} \lambda _1(K) \, \phi (x) = R \int _{\varOmega } K(x,y) \, \phi (y) \, dy, \end{aligned}$$

where $R=f^\prime (0)$. It has been shown in Lutscher et al. (2005) that $\lambda _1(K)$ is a strictly increasing function of $L$. If we assume $\int _{\varOmega } \phi (y) \, dy=1$ and integrate (11) over the habitat we obtain the following relation between $\lambda _1(K)$ and the dispersal success function $s(y)$:

12 $$\begin{aligned} \lambda _1(K) = R \int _{\varOmega } s(y) \, \phi (y) \, dy. \end{aligned}$$

**Fig. 3:**
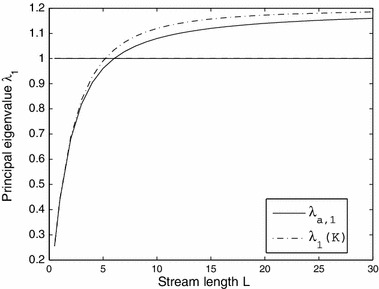
The principal eigenvalue $\lambda _1(K)$ and the dispersal success approximation $\lambda _{a,1}$ for linearization of (3) with $f'(0) = 1.2$ and asymmetric kernel $K$ with $D = 1$, $\beta = 1$, and $v = 0.1$. Note that $\lambda _{a,1}$ tends to underestimate $\lambda _1(K)$ as the domain length increases

Taking the approximation $\phi (x) \approx \frac{1}{L}$, we can estimate $\lambda _1(K)$ by

13 $$\begin{aligned} \lambda _1(K) \approx \lambda _{a,1}:= \frac{R}{L} \int _{\varOmega } s(y) \, dy \end{aligned}$$

which is known as the *dispersal success approximation*. To illustrate, Fig. 3 shows the dispersal success approximation (13) compared to the principal eigenvalue $\lambda _1(K)$ as a function of stream length for a sample kernel.

#### Including temporal variation

In this paper we consider a generalization of model (3) that allows for the kind of temporal variation in growth and dispersal that is found in stream ecosystems. We focus on including the inter-annual variations in growth and dispersal that arise from temporally fluctuating environments. An organism in such an environment experiences different growth and dispersal dynamics, depending on the year. In this case the model (3) becomes

14 $$\begin{aligned} n_{t+1} (x) = \int _{\varOmega } K_t(x,y) \, f_t(n_t(y)) \, dy \end{aligned}$$

where $K_t$ denotes the $t$th time step dispersal kernel and $f_t(n)$ the growth dynamics at time step $t$.

### Mathematical setting

We briefly review the mathematical setting and known results for population persistence in the context of integrodifference equations. We restrict our attention to the case where $K(x,y)=K(x-y)$ is a *difference kernel*, expressed in terms of the difference between the settling location $x$ and the starting point $y$. Although this includes the case of symmetric distance kernels where $K(x,y)= K(|x-y|)$ (such as (4)), we do not require symmetry since we are particularly interested in the case where $K$ is an asymmetric advective kernel such as (5). We assume the population densities are given by elements of $C(\varOmega )$, the Banach space of continuous real-valued functions defined on $\varOmega$. We first discuss the constant kernel case, then review some known results for temporally varying kernels.

#### Constant environments

We can rewrite Eq. (3) as

15 $$\begin{aligned} n_{t+1}(x)=F(n_t)(x) \end{aligned}$$

where $F:C(\varOmega ) \rightarrow C(\varOmega )$ is the nonlinear Hammerstein operator

16 $$\begin{aligned} F(n)(x)=\int _\varOmega K(x-y) f(n(y))\,dy. \end{aligned}$$

If $f$ and $K(x,y)$ are continuous, then it follows from the Arzelà–Ascoli theorem that $F$ is a compact operator. Moreover, since $K \ge 0$ and $f \ge 0$, the operator $F$ is a *positive* operator, mapping the cone of nonnegative functions $C_+(\varOmega )$ into itself.

The linearization of (15) at the equilibrium $n_*(x) = 0$ takes the form of a Fredholm equation of the first kind

17 $$\begin{aligned} n_{t+1}(x)=\fancyscript{L}(n_t)(x) = R \int _\varOmega K(x-y)n_t(y)\,dy \end{aligned}$$

where $\fancyscript{L}=F'(0)$ is the Fréchet derivative of $F$ at $n_*(x)=0$ and $R=f'(0)$ is the geometric growth rate for the population (Van Kirk and Lewis 1997). Since the Fréchet derivative of a compact operator is compact (Krasnoselskii 1964b), the operator $\fancyscript{L}$ is a compact bounded linear operator.

A further assumption regarding the positivity of $\fancyscript{L}$ allows one to connect the population dynamics of (15) with the spectral properties of $\fancyscript{L}$. Namely, we say the operator $\fancyscript{L}$ is *strongly positive* if for any continuous function $n \ge 0$ there exists a power $t = t(n)$ such that $\fancyscript{L}^t(n)(x) > 0$ for all $x \in \varOmega$. Biologically, this condition implies that on a connected habitat repeated application of the kernel will allow an individual that starts at any point $y \in \varOmega$ to eventually access all other points $x$ in $\varOmega$ (Lutscher and Lewis 2004, Condition A4). In this case, the Krein–Rutman theorem applies, and it follows that $\fancyscript{L}$ has a principal eigenvalue $\lambda _1 > 0$ such that $|\lambda | < \lambda _1$ for all other eigenvalues and $\lambda _1$ is the only eigenvalue associated with a positive eigenfunction $\phi$. Our assumptions on $K$ imply that the zero solution of (15) is linearly stable when $\lambda _1 < 1$ and unstable when $\lambda _1 > 1$. Moreover, for $\lambda _1 > 1$ there exists a nontrivial equilibrium solution of (15) (Hardin et al. 1990, see also Van Kirk and Lewis 1997 for the $L^2(\varOmega )$ case).

Within this context, the principal eigenvalue $\lambda _1$ is equal to the spectral radius of the operator $\fancyscript{L}$ which, by the Gelfand formula, can be expressed as

18 $$\begin{aligned} \lambda _1 = r(\fancyscript{L}) = \lim _{t \rightarrow \infty } \Vert \fancyscript{L}^t \Vert ^{1/t}= \inf _{t \ge 1} \Vert \fancyscript{L}^t \Vert ^{1/t} \end{aligned}$$

where

$$\begin{aligned} \Vert \fancyscript{L}\Vert = \sup _{\phi \in C(\varOmega )\backslash \{0\}} \frac{\Vert \fancyscript{L}\phi \Vert _\infty }{{\Vert \phi \Vert }_\infty } \end{aligned}$$

and $\Vert \cdot \Vert _\infty$ is the sup norm on $C(\varOmega )$ (Krasnoselskii 1964a, b). In particular, $\lambda _1 \le \Vert \fancyscript{L}\Vert .$ Moreover, if we consider a positive eigenfunction $\phi _1$ and integrate the associated equation

$$\begin{aligned} \lambda _1 \phi _1(x) = R \int _{\varOmega } K(x,y) \, \phi _1(y) \, dy, \end{aligned}$$

we have

19 $$\begin{aligned} \lambda _1\int _{\varOmega } \phi _1(y) \, dy = R \int _{\varOmega } s(y) \, \phi _1 (y) \, dy \le R \int _{\varOmega } \phi _1(y) \, dy, \end{aligned}$$

where we used the fact that $0\le s(y)\le 1$. Thus, $\lambda _1\le R = f'(0)$. If we assume there is dispersal loss from all points in the domain then $s < 1$ and we obtain a strict inequality

20 $$\begin{aligned} \lambda _1< R = f'(0). \end{aligned}$$

We can interpret (20) biologically as indicating that dispersal loss will reduce the growth rate below the intrinsic growth rate for the non-spatial model.

#### Temporally Varying Environments

A series of papers by Hardin et al. (1988a, b, 1990) consider the case where the Hammerstein operator is time-dependent so that

21 $$\begin{aligned} n_{t+1}(x)=F_t(n_t)(x) \end{aligned}$$

where

22 $$\begin{aligned} F_t(n)(x)=\int _\varOmega K_t(x-y) f(n(y))\,dy, \end{aligned}$$

with $K_t$ having parameters that are chosen randomly from some set (defining the allowable range of environmental conditions). Here the linearized operator $\fancyscript{L}_t=F'_t(0)$ is time-dependent, so the eigenvalue analysis discussed in the previous section does not apply. However, they show that population persistence can still be understood via the limit of operator norms

23 $$\begin{aligned} r = \lim _{t \rightarrow \infty } \Vert \fancyscript{L}_t \circ \cdots \circ \fancyscript{L}_2 \circ \fancyscript{L}_1\Vert ^{1/t} \end{aligned}$$

which acts as an effective spectral radius for the time-varying setting. Hardin et al. (1988a) derive conditions under which the limit (23) exists, and show how the quantity $r$ determines long-term population persistence or extinction for solutions of (21). The limit $r$ is similar to the dominant Lyapunov exponent (stochastic growth rate) of random matrix products, which has been applied to determine population persistence for a structured population in both correlated and uncorrelated random environments (e.g., Benaïm and Schreiber 2009) and coexistence for interacting structured populations living in a random environment (e.g., Roth and Schreiber 2014).

We consider (22) in the context of growth and dispersal in streams for the case of periodic and random dispersal parameters and show the Hardin et al. framework can be adapted to our setting. A range of different types of growth rates (and related elasticities) of populations have been introduced and used to study population dynamics in random environments (see e.g., Tuljapurkar 1990 and Tuljapurkar et al. 2003). In this paper we consider population persistence via an asymptotic growth rate

24 $$\begin{aligned} \varLambda := \lim _{t \rightarrow \infty } \left[ ~\int _\varOmega n_t(x) \, dx \right] ^{1/t}, \end{aligned}$$

where $n_t(x)$ is defined by the system

25 $$\begin{aligned} n_{t+1}(x)=\fancyscript{L}_t(n_t)(x) = \int _\varOmega K_t(x-y) f_t'(0) \, n_t(y)\,dy \end{aligned}$$

with nonzero initial condition $n_0(x) \ge 0$. We will prove that $\varLambda =r$, and hence, numerically, we can calculate $\varLambda$ to determine population persistence or extinction. We use this to consider several examples in the context of randomly fluctuating river populations.

Note that if $A$ is the infinitesimal generator of a continuous semigroup $T(t)=\{e^{tA}\}_{t\ge 0}$, the spectral bound of $A$ is defined as

$$\begin{aligned} s(A) = \sup \{\text {Re}(\lambda ) \ | \ \lambda \in \sigma (A)\}, \end{aligned}$$

where $\sigma (A)$ is the spectrum of $A$ (with $s(A) = -\infty$ if $\sigma (A) = \emptyset$) and the type (exponential growth bound) of the semigroup $T(t)$ is defined as

$$\begin{aligned} \omega _0 = \lim \limits _{t\rightarrow \infty } \frac{\log ||T(t)||}{t}. \end{aligned}$$

For many generators $A$ it is known that $-\infty \le s(A)\le \omega _0<\infty$ (see e.g., Hille and Phillips 1957) and the conditions for the equality of these two quantities have been studied (Greiner et al. 1981; Kato 1982; Thieme 2009). The quantities $s(A)$ and $\omega _0$ have also been used to derive persistence conditions for population models in temporally homogeneous or heterogeneous environments (see e.g., Thieme 2009). In this current work, $r$ and $\varLambda$, as defined by (23) and (24), are analogous to the spectral bound and type for the infinitesimal generator $A$ of a continuous semigroup $T$. Thus this work can be considered as a generalization of the idea of $s(A)=\omega _0$ and using such quantities to determine population persistence.

### Outline of the paper

In this paper, we study the integrodifference equation (15) for population persistence in temporally varying advective environments. In Sect. 2, we study the integro-difference equation with alternating kernels and growth rates in a periodically varying environment and obtain an explicit method to calculate the principal eigenvalue for the two-stage process. We also give the approximation of the principal eigenvalue by virtue of the dispersal success function and the redistribution function. In Sect. 3, we study the model in a randomly varying advective environment, where both the growth rate and the dispersal kernel are random. This contrasts with the earlier work by Hardin et al. (1988a), where only the growth rate fluctuated randomly. We derive the persistence metric $r$, similar to (23) and obtain its equivalence to the asymptotic growth rate (24). We also provide exact formula for the asymptotic growth rate when kernels take an asymmetric advective form (5) with randomly chosen parameters. This allows us to explicitly calculate population growth rates in randomly fluctuating river environments. Our various methods for calculating persistence and growth metrics are illustrated using numerical examples for models describing randomly fluctuating rivers. A short discussion completes the paper in Sect. 4.

## Alternating kernel model

We consider a deterministic case of time-varying kernels for the linearized model (17). In particular, we consider the case of alternating kernels $K_1(x,y)$ and $K_2(x,y)$ with associated growth rates $R_1$ and $R_2$. We can consider the two stages in succession using the linearized model (17) as:

$$\begin{aligned} n_{t+2} (x)&= R_2 \int _{\varOmega } K_2(x,y) \, n_{t+1}(y) \, dy \\&= R_2 \int _{\varOmega } K_2(x,y) \left[ R_1 \int _{\varOmega } K_1(y,z) \, n_t(z) \, dz \right] \, dy \\&= R_1 R_2 \int _{\varOmega } \int _{\varOmega } K_2(x,y) K_1(y,z) \, n_t(z) \, dz \, dy \\&= R_1 R_2 \int _{\varOmega } K(x,z) \, n_t(z)\,dz \\ \end{aligned}$$

where

26 $$\begin{aligned} K(x,z) = \int _{\varOmega } K_2(x,y) K_1(y,z) \, dy. \end{aligned}$$

In this way, the two-stage model can be considered as a single model (for two stages) via

27 $$\begin{aligned} n_{t+1} (x) = R \int _{\varOmega } K(x,y) \, n_t(y) \, dy, \end{aligned}$$

where $K$ is defined by (26) and $R=R_1 R_2$.

Let $\lambda _1$ be the principal eigenvalue associated with the two-stage model (27). The results in Sect. 1.3.1 imply the zero solution is unstable if $\lambda _1>1$ and it is stable if $\lambda _1<1$. Hence, the population persists if $\lambda _1>1$ and the population will be extinct if $\lambda _1<1$. It follows from estimate (20) that

28 $$\begin{aligned} \lambda _1 \le R_1 R_2. \end{aligned}$$

Since the principal eigenvalue $\lambda _1$ for the two-stage process models two years of population dynamics, the quantity $\sqrt{\lambda _1}$ would be an effective single year growth estimate. In this sense, estimate (28) says the effective annual growth rate is bounded above by the geometric mean of the two growth rates.

We can gain further insight into the two-stage $\lambda _1$ by working directly with the eigenfunction equation. Suppose $\lambda \ne 0$ is an eigenvalue associated with an eigenfunction $\phi$ for (27). Then

29 $$\begin{aligned} R_1 R_2 \int _{\varOmega } K(x,y) \, \phi (y) \, dy = \lambda \, \phi (x), \end{aligned}$$

where $K$ is the two-step kernel (26). Let $\psi$ be defined by

30 $$\begin{aligned} \psi (x) = R_1 \int _{\varOmega } K_1(x,y) \, \phi (y) \, dy. \end{aligned}$$

Then $\phi$ solves (29) if and only if

31 $$\begin{aligned} \phi (x) = \frac{R_2}{\lambda } \int _{\varOmega } K_2(x,y) \, \psi (y) \, dy. \end{aligned}$$

Now suppose $K_1$ and $K_2$ are advective dispersal kernels of the form (5) for some $\beta _1, D_1,v_1$ and $\beta _2, D_2, v_2$. Differentiating (30) and using (8) for $K_1$ we have

32 $$\begin{aligned} \psi ''(x) = \frac{v_1}{D_1} \psi '(x) + \frac{\beta _1}{D_1} \psi (x) - \frac{\beta _1}{D_1} R_1 \, \phi (x). \end{aligned}$$

Similarly, differentiating (31) and using (8) for $K_2$ we have

33 $$\begin{aligned} \phi ''(x) = \frac{v_2}{D_2} \phi '(x) + \frac{\beta _2}{D_2} \phi (x) - \frac{\beta _2}{D_2} \frac{R_2}{\lambda } \, \psi (x). \end{aligned}$$

It follows that the eigenfunction $\phi$ solves the fourth order equation

34 $$\begin{aligned} \phi ^{(4)} - B \phi ^{(3)} - \left( \frac{\beta _1}{D_1} + \frac{\beta _2}{D_2} - \frac{v_1 v_2}{D_1 D_2} \right) \phi ^{(2)} + C \phi ' + \frac{\beta _1 \beta _2}{D_1 D_2} \left( 1 - \frac{R_1 R_2}{\lambda } \right) \phi = 0,\nonumber \\ \end{aligned}$$

where

$$\begin{aligned} B = \left[ \frac{v_1}{D_1} + \frac{v_2}{D_2} \right] \qquad \text { and } \qquad C = \left[ \frac{v_1 \beta _2 + \beta _1 v_2}{D_1 D_2} \right] . \end{aligned}$$

The boundary conditions can be determined as follows. Suppose $\varOmega = [0,L]$. First, differentiating (30) and (31) and using the definition of $K$ we have

35 $$\begin{aligned} \phi '\left( 0 \right)&= a_{1,2} \, \phi \left( 0 \right) \end{aligned}$$

36 $$\begin{aligned} \phi '\left( L \right)&= a_{2,2} \, \phi \left( L \right) \end{aligned}$$

and

37 $$\begin{aligned} \psi '\left( 0 \right)&= a_{1,1} \, \psi \left( 0 \right) \end{aligned}$$

38 $$\begin{aligned} \psi '\left( L \right)&= a_{2,1} \, \psi \left( L \right) \end{aligned}$$

where $a_{i,j}$ denotes the constant $a_i$ in (5) for the kernel $K_j$. Next, differentiating (33), using (37)–(38) and (33) we obtain two additional boundary conditions:

39 $$\begin{aligned} \phi '''\left( 0 \right) \!&= \! \left( a_{1,1} + \frac{v_2}{D_2}\right) \phi ''\left( 0 \right) \!-\! a_{1,1} \frac{v_2}{D_2} \phi '\left( 0 \right) \!+\! \frac{\beta _2}{D_2} \left( a_{1,2} \!-\! a_{1,1} \right) \phi (0), \end{aligned}$$

40 $$\begin{aligned} \phi '''\left( L \right) \!&= \! \left( a_{2,1} \!+ \!\frac{v_2}{D_2} \right) \phi ''\left( L \right) \!-\! a_{2,1} \frac{v_2}{D_2} \phi '\left( L \right) \!+\! \frac{\beta _2}{D_2} \left( a_{2,2} \!-\! a_{2,1} \right) \phi (L). \end{aligned}$$

The differential equation (34) together with the four boundary conditions (35)–(36) and (39)–(40) comprise a fourth-order boundary-value problem for the eigenpair ($\lambda ,\phi )$ of (29) in the case of two-stage advective kernels. The general solution of equation (34) has the form

41 $$\begin{aligned} \phi (x) = c_1 e^{r_1 x} + c_2 e^{r_2 x} + c_3 e^{r_3 x} + c_4 e^{r_4 x} \end{aligned}$$

where $r_i \in {\mathbb {C}}$ are the roots of the associated characteristic polynomial. Applying the boundary conditions to $\phi$ yields a fourth-order linear system of the form $A \varvec{c} = \varvec{0}$ for the coefficients $\varvec{c} = [ c_1 \ c_2 \ c_3 \ c_4 ]^T$. This system admits a nontrivial solution, and hence $\phi$ in (41) is nontrivial, only if $\det A = 0$, which, for a fixed domain length $L$, defines an implicit equation for $\lambda$. Solving $\det A = 0$, we then obtain the principal eigenvalue $\lambda _1 = \lambda _{1,\mathrm{twostep}}$ of the two-stage operator. To illustrate, we derive the fourth order boundary value problem for (27) in the symmetric kernel case in Appendix A.1. We also note that this process can be used to determine the critical domain length by setting $\lambda = 1$ and determining conditions on $L$ for which the fourth-order system admits a nontrivial solution (see e.g., Jin and Lewis 2011).

### *Example 1*

To illustrate how the principal eigenvalue $\lambda _{1,\mathrm{twostep}}$ of (29) depends on flow velocities we consider the case of variable flow rates $v_1$ and $v_2$, but with fixed mean $\bar{v} = (v_1 + v_2)/2$ for a habitat $\varOmega =[0,L]$ with $L=20$, $R_1=1.2$, $R_2=1.5$, $D_1=D_2=1$, $\beta _1=\beta _2=1$.

**Fig. 4:**
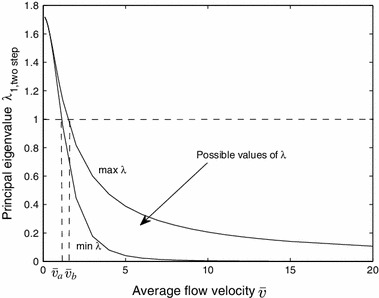
The relationship between the principal eigenvalue of (29) for the alternating kernel model and average flow velocity $\bar{v} = (v_1 + v_2)/2$. Parameters are: $R_1 = 1.2$, $R_2 = 1.5$, $D_1 = 1$, $D_2 = 1$, $\beta _1 = 1$, $\beta _2 = 1$, $\varOmega =[0,L]$ with $L=20$

As the flow rates $v_1$ and $v_2$ vary, while keeping $\bar{v}$ fixed, the value of $\lambda _{1,\mathrm{twostep}}$ varies within some interval. Figure 4 shows the possible values of $\lambda _{1,\mathrm{twostep}}$ as a function of the average of flow velocity $\bar{v} \in [0,20]$. Overall, $\lambda _{1,\mathrm{twostep}}$ decreases with $\bar{v}$. When the average $\bar{v}$ is sufficiently small (i.e., $\bar{v} <\bar{v}_a$), then $\lambda _{1,\mathrm{twostep}}>1$ regardless of $v_1$ and $v_2$, which corresponds to persistence of the population; when $\bar{v}$ is sufficiently large (i.e., $\bar{v} >\bar{v}_b$), then $\lambda _{1,\mathrm{twostep}}<1$ regardless of $v_1$ and $v_2$, which corresponds to extinction. For moderate values of $\bar{v}$ (i.e., $\bar{v}_a<\bar{v} <\bar{v} _b$), different combinations of $v_1$ and $v_2$ may lead to $\lambda _{1,\mathrm{twostep}}>1$ or $\lambda _{1,\mathrm{twostep}}<1$, and hence, the population can persist or go extinct in different fluctuating flows even though the mean of flow velocity is constant.

**Fig. 5:**
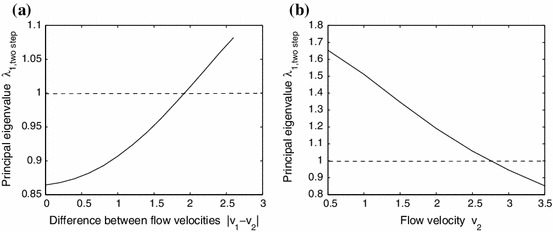
**a** The relationship between the principal eigenvalue for (29) for the alternating kernel model and the difference of $v_1$ and $v_2$ while keeping the average flow fixed at $\bar{v} =(v_1+v_2)/2=1.3$. **b** The relationship between the principal eigenvalue for (29) for the two-stage model and $v_2$ where $v_1=0.1$. The other parameters are: $R_1 = 1.2$, $R_2 = 1.5$, $D_1 = D_2 = 1$, $\beta _1 = \beta _2 = 1$, and $\varOmega =[0,L]$ with $L=20$

For a fixed mean $\bar{v}$, we can also consider $\lambda _{1,\mathrm{twostep}}$ as a function of the variation in flow $|v_1 - v_2|$. Figure 5a illustrates this for the case $\bar{v} = 1.3$. Note that $\lambda _{1,\mathrm{twostep}}$ is an increasing function of $|v_1-v_2|$. For this average flow velocity, the smaller the variation between $v_1$ and $v_2$, the smaller the possibility that the population can persist in the river.

If we fix $v_1$ and vary only $v_2$, then Fig. 5b shows that $\lambda _{1,\mathrm{twostep}}$ is a decreasing function of $v_2$. This coincides with the fact that, when the flow velocity in one step is constant, then the larger the flow velocity in the second step, the harder it is for the population to persist in the river.

### *Example 2*

**Fig. 6:**
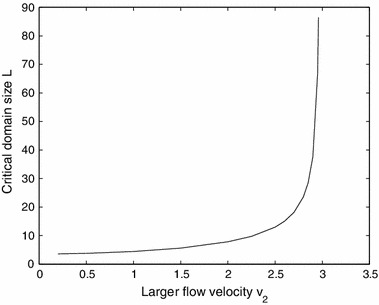
The relation between the critical domain size and $v_2$ for the alternating kernel model with $v_1=0.1$, $R_1 = 1.2$, $R_2 = 1.5$, $D_1 = D_2 = 1$, and $\beta _1 = \beta _2 = 1$. As $v_2\rightarrow 3$, the critical domain size approaches infinity

To illustrate how one can study critical domain size questions in this setting we consider the case of fixing $$v_1 = 0.1$$ and determining the critical domain length as a function of $$v_2$$ (leaving the other parameters as in Example 1). We can study this by setting $$\lambda = 1$$ in (34) and determining conditions on $$L$$ for which the fourth-order system admits a nontrivial solution. An example is shown in Fig. 6. As one might expect, as $$v_2$$ increases the critical domain length increases, with $$L $$ approaching infinity as $$v_2$$ tends to some value. Since the critical domain size represents the minimal length of the river such that population can persists, this observation implies that the higher the flow the more difficult it is for the population to persist in the river, consistent with earlier results in Lutscher et al. (2005) and Jin and Lewis (2011).

Finally, we note that, similar to the dispersal success approximation in (13), we can use redistribution and dispersal success to approximate $\lambda _{1,\mathrm{twostep}}$ as

42 $$\begin{aligned} \lambda _{1,\mathrm{twostep}} \approx \lambda _{a,12}: = \frac{R_1 R_2}{L} \int _{\varOmega } s_2(y) \, r_1(y) \, dy, \end{aligned}$$

where $r_1$ is the redistribution function corresponding to $K_1$ and $s_2$ is the dispersal success function corresponding to $K_2$. In Appendix A.2 we include an example comparing this approximation with $\lambda _{1,\mathrm{twostep}}$ for different domain lengths $L$.

The differential equation approach in this section for alternating kernels can be generalized to the case of a sequence of $n$ kernels (Jacobsen and McAdam 2014). However, we will instead turn to the case of random kernels, and in particular, asymmetric Laplace kernels whose parameters are chosen from a given distribution.

## Random kernel model

In this section, we consider the model

43 $$\begin{aligned} n_{t+1}(x) =F_t(n_{t})(x) = \int _{\varOmega } K_t(x-y) f_t(n_{t}(y)) \, dy, \end{aligned}$$

where $f_t$ and $K_t$ denote “random” growth and dispersal kernels at step $t$. We apply the theory of Hardin et al. (1988a, b, 1990) to the random difference kernel model (43) and show that the long-term population persistence or extinction can be determined by the generalized spectral radius $r$ defined by (23). We also define another quantity $\varLambda$ (as in (24)), which is mathematically equivalent to $r$, is computationally easier to work with, and most importantly has a biological meaning of the asymptotic growth rate of the population. We use this alternate framework to consider several examples for random kernels.

### Persistence metrics

First, for notational clarity, we rewrite (43) as

44 $$\begin{aligned} n_{t+1}(x)=F_{\alpha _t}(n_{t})(x) := \int _\varOmega K_{\alpha _t}(x-y)f_{\alpha _t}(n_{t}(y))\,dy \end{aligned}$$

where $\{\alpha _t\}_{t\ge 0}$ is a sequence of independent identically distributed random variables taking values in an index set ${\mathcal {A}}$ (representing the range of environmental conditions).

We make the following assumptions on the kernels $K_{\alpha _t}$ and growth functions $f_{\alpha _t}$:

1.
  1. For each $\alpha \in {\mathcal {A}}$, $K_\alpha (x-y)$ is continuous for $x,y \in \varOmega$.
  2. There exists constants $\underline{K}> 0$ and $\overline{K}$ such that
    $$\begin{aligned} \underline{K}\le K_\alpha (x-y)\le \overline{K} \quad \text { for all } \alpha \in \mathcal {A} \text { and } x, y\in \varOmega . \end{aligned}$$
2.
  1. For any $\alpha \in {\mathcal {A}}$, $f_\alpha :\mathbb {R}\rightarrow [0,\infty )$ is continuous with $f_\alpha (u)=0$ for all $u\le 0$.
  2. There exists $m> 0$, $\underline{f}>0$, and $\overline{f}>0$, such that for any $\alpha \in \mathcal {A}$,
    - $f_\alpha (u)$ is an increasing function in $u$.
    - $$0\le f_\alpha (u)\le m$$ for all $$u\in C_+(\varOmega )$$.
    - If $$0 < v < u$$ then $$\frac{f_\alpha (u)}{u}<\frac{f_\alpha (v)}{v}$$.
    - $f_\alpha$ is right differentiable at $0$. For simplicity, we denote the right derivative as $f_\alpha ^\prime (0)$.
    - $\frac{f_\alpha (u)}{u}\rightarrow f_\alpha ^\prime (0)$ as $u\rightarrow 0^+$, uniformly for $\alpha \in \mathcal {A}$
    - $$ \underline{f}=\inf \limits _{\alpha \in \mathcal {A}}f_\alpha ^\prime (0)\le f_\alpha ^\prime (0)\le \overline{f}$$
    - For $b=m \overline{K} |\varOmega |$, there exists $\underline{f_1}=\inf \limits _{\alpha \in \mathcal {A}}f_\alpha (b)>0$.
3. There exists $\alpha ^*\in \mathcal {A}$ such that $F_{\alpha }(u)\le F_{\alpha ^*}(u)$ for all $\alpha \in \mathcal {A}$ and $u\in C_+(\varOmega )$.

Under these assumptions it can be shown (see Appendix A.3) that the framework of Hardin et al. (1988a) can be applied to the nonlinear model (44), which yields the following result:

#### **Theorem 1**

Assume that $F_{\alpha _t}$ ($\alpha _t\in \mathcal {A}$ for $t\in \mathbb {N}$) defined by (44) satisfies (C1)–(C3). For nonzero initial data $n_0\in C_+(\varOmega )$, the population $n_t$ of (44) converges in distribution to a stationary distribution $\mu ^*$, independent of $n_0$, that is either concentrated at $0 \in C_+(\varOmega )$ (extinction) or supported in $C_+(\varOmega )\backslash \{0\}$ (persistence).

Moreover, let

45 $$\begin{aligned} r=\lim \limits _{t\rightarrow \infty }\Vert \fancyscript{L}_{\alpha _t}\circ \cdots \circ \fancyscript{L}_{\alpha _1}\Vert ^{1/t}, \end{aligned}$$

where $\alpha _t\in \mathcal {A}$ for all $t\ge 1$ and $\fancyscript{L}_{\alpha _t}:=F_{\alpha _t}^\prime (0)$ is the linearization of $F_{\alpha _t}$ at $n=0$. The following results hold:

- If $r<1$, then the population will go extinct.
- If $r>1$, then the population will persist.

The quantity (45) provides a means to study population persistence for our integrodifference stream model in the context of random dispersal and growth, within the framework of the hypotheses (C1)–(C3). We now show there is an alternate metric for (44), which can also be used to analyze persistence of the population, is numerically easier to work with, and has a clear biological interpretation.

Consider the linearization of (44) at the trivial solution

46 $$\begin{aligned} n_{t+1}(x)= R_{\alpha _t} \int _{\varOmega } K_{\alpha _t}(x-y) \, n_{t}(y) \, dy, \end{aligned}$$

where $R_{\alpha _t} = f_{\alpha _t}^{\prime }(0)$. Let $n_t(x)$ be the solution of (46) for initial value $n_0\in C_+(\varOmega )\backslash \{0\}$. Then the average growth rate of the population over the first $t$ steps can be written as

$$\begin{aligned} \text{ Average } \text{ growth } \text{ rate } \text{ over } \text{ first } t \text{ steps }= \left[ \frac{ \int _{\varOmega } n_t(x) \, dx }{\int _{\varOmega } n_0(x) \, dx}\right] ^{1/t}. \end{aligned}$$

Define the limit of this average as

47 $$\begin{aligned} \varLambda := \lim _{t \rightarrow \infty } \left[ \frac{ \int _{\varOmega } n_t(x) \, dx }{\int _{\varOmega } n_0(x) \, dx}\right] ^{1/t}. \end{aligned}$$

In this sense, the limiting constant $\varLambda$ represents the asymptotic growth rate of the population.

In Appendix A.4 we show the limit in (47) exists and is independent of the initial function $n_0\in C_+(\varOmega )\backslash \{0\}$, so the definition in (47) can be simplified to

48 $$\begin{aligned} \varLambda = \lim _{t \rightarrow \infty } \left[ ~\int _{\varOmega } n_t(x) \, dx \right] ^{1/t} \end{aligned}$$

for any $n_0\in C_+(\varOmega )\backslash \{0\}$. Furthermore, as we state in the theorem below, the asymptotic growth rate $\varLambda$ and the constant $r$ are equal. The proof of this theorem, provided in Appendix A.4, also includes the proof of the existence of the limit in (47) and the equivalence of (47) and (48).

#### **Theorem 2**

Let $r$ and $\varLambda$ be defined in (45) and (48), respectively. Then $\varLambda = r$.

#### *Remark 1*

It follows from Theorems 1 and 2 that if $\varLambda >1$, the population will be persistent and if $\varLambda <1$, the population will go extinct. Thus population persistence or extinction can be studied by computing $\varLambda$ for the iterates $n_t$ of the linear model (46) (recalling $R_{\alpha _t}$ and $K_{\alpha _t}$ change at each step).

We illustrate applications of Theorem 2 for several examples of (46), using $\varLambda$ to determine population persistence or extinction. For simplicity, we use (48) to approximate $\varLambda$.

#### *Example 3*

(Random two kernel model) Consider (46) where the kernel $K_t$ is chosen at random from one of two asymmetric advective kernels $K_1$ and $K_2$, with equal probability. For $K_i$ as in (5), we assume $v_1=0.1, v_2=1,$ $D_1 = D_2 = 1$, and $\beta _1 = \beta _2 = 1$. Since we are effectively flipping a coin to determine the kernel $K_t$ we call this the “coin-flip kernel model” or CFK model. We assume $R_t = 1.2$ if $K_t = K_1$ and $R_t = 1.5$, if $K_t = K_2$.

**Fig. 7:**
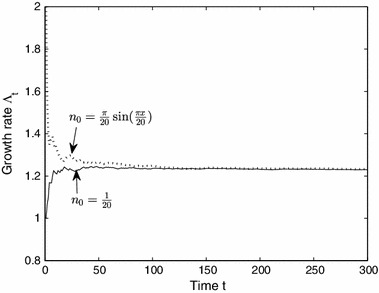
Plot of $\varLambda _t$ for the CFK model illustrating the rate of convergence and independence of $\varLambda$ on the initial state $n_0$. The parameters are: $\varOmega =[0,L]$ with $L=20$, $D_1 = D_2 = 1$, $\beta _1 = \beta _2 = 1$, flow rate $v_1=0.1, v_2=1$ (chosen with equal probability) and $R_1 = 1.2$ (when $v=v_1$), and $R_2 = 1.5$ (when $v=v_2$). The convergence rate appears roughly equal for each initial state

First, in Fig. 7 we illustrate sample rates of convergence for $\varLambda$ for different initial conditions by plotting the $t\mathrm{th}$-approximation of $\varLambda$ defined by (48)

49 $$\begin{aligned} \varLambda _t:= \left( ~ \int _{\varOmega } n_t(x) \, dx \right) ^{1/t} \end{aligned}$$

for two different initial states $n_0 = 1/20$ and $n_0=(\pi /20) \sin (\pi x/20)$, on a habitat $\varOmega = [0,20]$. Notice $\varLambda _t$ converges to the same value of $\varLambda \approx 1.22$ for each initial state and at roughly the same rate.

**Fig. 8:**
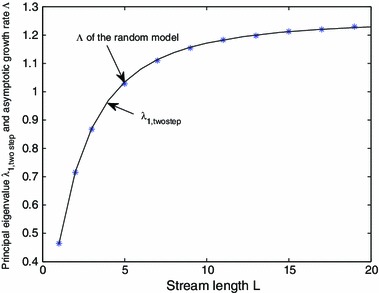
Comparison of the principal eigenvalues of the alternating kernel model (*solid line*) with $\varLambda$ for the random CFK model (*stars*). The $\varLambda$ values are approximated by $\varLambda _t$ for large $t$ defined in (49), while the principal eigenvalue for the alternating kernel model are obtained by solving the boundary-value problem (34)–(40). The parameters are: $D_1 = D_2 = 1$, $\beta _1 = \beta _2 = 1$, $v_1=0.1, v_2=1$ and $R_1 = 1.2$ (when $v=v_1$), and $R_2 = 1.5$ (when $v=v_2$)

Next, we compare the principal eigenvalue for the alternating kernel model from Sect. 2 with the value of $\varLambda$ for the random CFK model. Figure 8 shows a plot of the principal eigenvalue $\lambda _{1,\mathrm{twostep}}$ of the alternating kernel model (27) (using the same parameters from the CFK model) with $\varLambda$ for the the CFK model (46). The principal eigenvalue of the alternating kernel model appears to match well with $\varLambda$ for the random model.

#### *Example 4*

(Log-normal flow velocities) Our next example considers (46) with the flow rate for kernel $K_t$ chosen from a log-normal distribution (keeping the other parameters fixed). We consider the relation between the asymptotic growth rate $\varLambda$ as a function of the variance in flow rate, while maintaining a fixed mean.

**Fig. 9:**
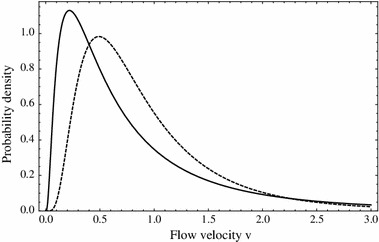
Probability density functions for a log-normal distribution with fixed mean $\bar{v}=0.95$ and variance $\nu =1.5$ (*solid*) and $0.5$ (*dashed*). Note that the occasional higher flow values for the higher variance case are balanced by more frequent lower flow rates in order to keep the mean fixed

First, to illustrate the log-normal distribution, Fig. 9 shows the probability density function for a log-normal distribution with a fixed mean for two different variances.

**Fig. 10:**
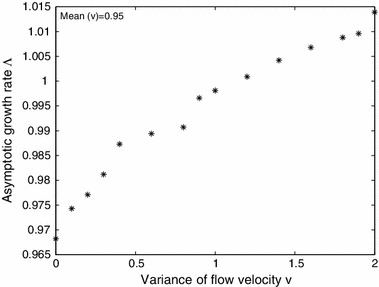
Plot of asymptotic growth rate $\varLambda$ for (46) vs. the variance of the flow velocity. The flow velocities in $K_t$ are chosen from a log-normal distribution with mean fixed at 0.95. The other parameters are held constant at $R = 1.2$, $D= 1$, $\beta = 1$ and $\varOmega =[0,L]$ with $L=20$. The values of $\varLambda$ are approximated by plotting $\varLambda _t$ for large $t$

Figure 10 shows an example of how the asymptotic growth rate $\varLambda$ depends on the variance of flow velocity, assuming a fixed mean. We see that $\varLambda$ tends to increase as the variance increases. For a single step kernel with mean flow velocity $v = 0.95$, the associated principal eigenvalue $\lambda _1 < 1$, corresponding to extinction. However, if we increase the variance while keeping the mean fixed, the higher flow velocities are balanced by more frequent low flow velocities which provide favorable conditions for survival. The values of $\varLambda$ are estimated by computing $\varLambda _t$ for $t>\!>1$. We note that the numerical results are identical for $\varOmega =[0,L]$ and $\varOmega =[-L/2,L/2]$.

**Fig. 11:**
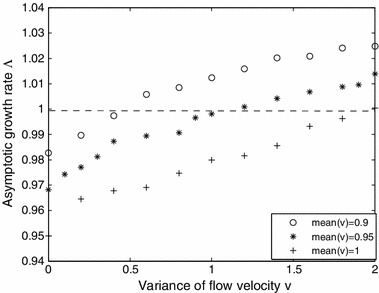
Plot of asymptotic growth rate $\varLambda$ for (46) vs. the variance of the flow velocity for three different fixed means. The flow velocities in $K_t$ are chosen from a log-normal distribution with means fixed at $0.9, 0.95,$ and $1$. The other parameters are held constant at $R = 1.2$, $D= 1$, $\beta = 1$, and $\varOmega =[0,L]$ with $L=20$. The values of $\varLambda$ are approximated by plotting $\varLambda _t$ for large $t$

Figure 11 shows how $\varLambda$ depends on the variance $\nu$ of the log-normal flow rate $v$ for three different fixed means $\mu = 0.9$, $0.95$ and $1$. Consistent with what one might expect, the higher the average flow rate the smaller $\varLambda$ is, and hence, the harder it is for the population to persist. Again, the values of $\varLambda$ are estimates based on $\varLambda _t$ for large $t$.

### Explicit calculation for $\varLambda _t$

In this section we compute an exact formula for the approximation $\varLambda _t$ of the asymptotic growth rate $\varLambda$. For notational simplicity, we write $K_{\alpha _t}$ as $K_t$ and $R_{\alpha _t}$ as $R_t$. Beginning with a uniform initial density $n_0(x) = 1$ on the habitat $\varOmega$, we have

$$\begin{aligned} \varLambda _{1}&= \int _{\varOmega } n_1(x) \, dx = \int _{\varOmega } \left( R_1 \int _{\varOmega } K_1(x,y) \, dy \right) \, dx = R_1 \int _{\varOmega } \int _{\varOmega } K_1(x,y) \, dx \, dy \\&= R_1 \int _{\varOmega } s_1(y) \, dy, \end{aligned}$$

where $s_1(y)$ is the dispersal success function for the kernel $K_1$. Similarly,

$$\begin{aligned} \varLambda _{2}&= \left( \int _{\varOmega } \left( R_2 \int _{\varOmega } K_2(x,y) \, n_1(y) \, dy \right) \, dx \right) ^{1/2} \\&= \left( R_1 R_2 \int _{\varOmega } \int _{\varOmega } \int _{\varOmega } K_2(x,y) \, K_1(y,z) dz \, dy \, dx \right) ^{1/2} \\&= \left( R_1 R_2 \int _{\varOmega } \int _{\varOmega } K_2(x,y) \, r_1(y) \, dy \, dx \right) ^{1/2} \\&= \left( R_1 R_2 \int _{\varOmega } s_2(y) \, r_1(y) \, dy \right) ^{1/2} \end{aligned}$$

where $s_2(y)$ is the dispersal success function for $K_2$ and $r_1(y)$ is the redistribution function for $K_1$. Continuing, we have

50 $$\begin{aligned} \varLambda _{3}&= \left( R_1 R_2 R_3 \int _{\varOmega } \int _{\varOmega } s_3(y) \, K_2(y,z) \, r_1(z) \, dy \, dz \right) ^{1/3} \end{aligned}$$

51 $$\begin{aligned} \varLambda _{4}&= \left( R_1 R_2 R_3 R_4 \int _{\varOmega } \int _{\varOmega } \int _{\varOmega } s_4(z_3) \, K_3(z_3,z_2) \, K_2(z_2 , z_1) \, r_1(z_1) \, dz_3 \, dz_2 \, dz_1 \right) ^{1/4}\quad \quad \end{aligned}$$

and, in general, for $t > 3$ we have

52 $$\begin{aligned} \varLambda _{t} = \fancyscript{R}_t^{1/t} \, M_t^{1/t} \end{aligned}$$

where $$\fancyscript{R}_t = R_1 R_2 \cdots R_t$$ and

53 $$\begin{aligned} M_t&= \underbrace{\, \int _{\varOmega } \cdots \int _{\varOmega } \, }_{t-1 \ \mathrm{terms}} s_{t}(z_{t-1}) \, \prod _{i=2}^{t-1} K_{i}(z_{i},z_{i-1}) \, r_1(z_1) \, dz_{t-1} \ldots \,dz_1 \end{aligned}$$

54 $$\begin{aligned}&= \underbrace{\, \int _{\varOmega } \cdots \int _{\varOmega } \, }_{t+1 \ \mathrm{terms}} \, \prod _{i=1}^{t} K_{i}(z_{i},z_{i-1}) \, dz_{t} \ldots dz_0. \end{aligned}$$

We can compute this exactly when the kernels $K_i$ are advective kernels (5) with random parameters $v_i, \beta _i$ and $D_i$, provided no two sets of kernels parameters repeat themselves exactly (which is reasonable in the case of random parameter values). Roughly speaking, each population stage will be represented by a sum of exponentials, although the number of terms grows at each step. More precisely, for $t \in \mathbb {N}$

55 $$\begin{aligned} n_t(x) = \fancyscript{R}_t \, \left( {\rho }_{0,t} + \sum _{j=1}^{t} {\rho }_{a_j,t} \, e^{a_j x} + \sum _{j=1}^{t} {\rho }_{b_j,t} \, e^{b_j x} \right) \end{aligned}$$

where $a_j := a_1$ and $b_j := a_2$ in definition (5) for kernel $K_j$, and ${\rho }_{r,t}$ are certain computable coefficients that depend on the kernels up to step $t$ (their precise form is interesting but not essential so we present them in Appendix A.5). If $\varOmega = (-L/2,L/2)$, then by integrating Eq. (55) on $\varOmega$ we obtain an exact expression for $\varLambda _t$:

56 $$\begin{aligned} \varLambda _t = \fancyscript{R}_t^{1/t} \left[ L \, {\rho }_{0,t} + 2 \sum _{j=1}^{t} \left( \frac{{\rho }_{a_j,t}}{a_j} \sinh \frac{ a_j L}{2} + \frac{{\rho }_{b_j,t}}{b_j} \sinh \frac{ b_j L}{2} \right) \right] ^{1/t}. \end{aligned}$$

**Fig. 12:**
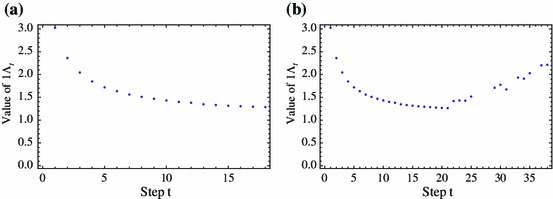
Calculations of $\varLambda _t$ from (56) for the case of a lognormal distribution with mean 0.95 and variance 1.5 (remaining setup as in Example 4). **a** $t = 1,\dots 20$; the data shows a reasonable trend, **b** $t=1,\dots 40$; after about $t=20$ the results are simply noise due to error accumulation

Although (56) provides an exact formula for $\varLambda _t$ for the random kernel case, it is not particularly stable for numerical calculations due to error accumulation in light of the many small divisors that appear in the coefficients ${\rho }_{r,t}$ (e.g., see (72)–(74) in Appendix A.5). This issue is compounded by the fact that our previous calculations of $\varLambda _t$ via numerical integration of (48) showed that the convergence to $\varLambda$ is fairly slow (e.g., see Fig. 7). Figure 12 illustrates an application of (56) for a specific case of Example 4 with lognormal flow velocities.

## Discussion

Even though classical ecological models assume environmental uniformity, the true natural environment shows a high degree of temporal variability. While the yearly specifics of the environmental variations rarely can be predicted, the general nature of the variability, as measured over many years, can be described statistically. One emerging challenge in mathematical biology has been to incorporate such measures of environmental variability into mathematical models for population persistence (see, for example, Benaïm and Schreiber 2009; Tuljapurkar 1990; Tuljapurkar et al. 2003; Schreiber 2010; Roth and Schreiber 2014). Although much recent mathematical attention has focused on this challenge, the pioneering work by Hardin et al. (1988a) actually provided mathematical tools to understand variability for integrodifference equations, as long as a quarter of a century ago.

While the mathematical foundation of our work rests on the seminal papers by Hardin et al. (1988a, b), Hardin et al. (1990), the methods we developed here have extend the approach significantly, and also transform the rather abstract results into concrete applications for the dynamics of river populations. Specifically, and perhaps most importantly, we have connected the Hardin et al. (1988a) metric $r$ (45) to a more biologically reasonable equivalent metric, the asymptotic growth rate of the linearized operator, $\varLambda$ (48). Indeed, we established the mathematical equivalence of $r$ and $\varLambda$ and interpreted this equivalence in terms of equivalent persistence metrics for the underlying stochastic, nonlinear dynamical system (44). Recall that $r$ and $\varLambda$ are defined as

$$\begin{aligned} r=\lim \limits _{t\rightarrow \infty }\Vert \fancyscript{L}_{\alpha _t}\circ \cdots \circ \fancyscript{L}_{\alpha _1}\Vert ^{1/t}, \end{aligned}$$

where $\fancyscript{L}_{\alpha _t}$ maps from $C(\varOmega )$ to $C(\varOmega )$, and

$$\begin{aligned} \varLambda := \lim _{t \rightarrow \infty } \left[ ~{ \int _{\varOmega } n_t(x) \, dx }\right] ^{1/t}. \end{aligned}$$

We can rewrite $\varLambda$ as

$$\begin{aligned} \varLambda =\lim _{t \rightarrow \infty } \left[ { ||n_t||_{L^1(\varOmega )} }\right] ^{1/t}=\lim _{t \rightarrow \infty } \left[ { ||\fancyscript{L}_{\alpha _t}\circ \cdots \circ \fancyscript{L}_{\alpha _1}(n_0)||_{L^1(\varOmega )} }\right] ^{1/t}, \end{aligned}$$

which yields $\varLambda =\lim \nolimits _{t\rightarrow \infty }\Vert \fancyscript{L}_{\alpha _t}\circ \cdots \circ \fancyscript{L}_{\alpha _1}\Vert ^{1/t}$, but in this case each $\fancyscript{L}_{\alpha _t}$ is considered to map from $L^1(\varOmega )$ to $L^1(\varOmega )$. Therefore, we can interpret our result mathematically as stating that, when studying population persistence for our random model (44), it does not matter whether the function space is chosen as $C(\varOmega )$ or $L^1(\varOmega )$. However, this result extends beyond the rather narrow mathematical interpretation given above; the asymptotic growth rate $\varLambda$ has more biological significance and can be easier to calculate than $r$.

The connection between $r$ and $\varLambda$ has allowed us to infer persistence properties of the nonlinear stochastic dynamical system, describing population growth and dispersal in rivers, based on $\varLambda$. In particular, it means that our explicit calculations of the asymptotic growth rate for river systems with asymmetric exponential dispersal (56) can be rigorously connected to persistence in the associated nonlinear system.

In our analysis we also developed a connection between periodically fluctuating river system, with asymmetric exponential dispersal, and a differential operator describing growth of an associated eigenfunction. Numerical results show a close concordance of persistence thresholds for the *alternating kernel model*, where good and bad years alternate, and those for a related *coin flip kernel model*, where good and bad years are chosen randomly with equal probability (Fig. 8).

The class of models in this paper can be generally applied to river or stream populations, where unidirectional flow dominates. However, particular stream populations are likely to require more detailed and specific models. One advantage of a general model is the ability to draw general conclusions. What can be concluded, in general, from the models in this paper regarding the role of variability in persistence in streams and rivers? First, longer streams (Fig. 3) and lower flow rates (Figs. 4, 5b) increase the likelihood of persistence, and higher flow streams must be longer, providing more habitat, if populations are to persist (Fig. 6). These, by themselves are not new theoretical results, and have been understood theoretically since the work of Speirs and Gurney (2001). However, a closer look at Fig. 4 shows that the variability in the flow velocity, as given in the alternating kernel model, can determine persistence outcomes as much as the mean velocity. Specifically, increased variability gives an increased probability of persistence (Fig. 5a). Here the effects of flow rate variation do not simply average out, and the beneficial effect of a low-flow period more than compensates for the detrimental effect of a corresponding high-flow period. This relationship between flow variability and persistence holds over to the more complex case where the dispersal kernel is chosen from a family where the flow velocity is drawn from a continuous probability density function, such as a log-normal distribution (Figs. 9, 10, 11). We considered variations of the parameters in the positive space for the two-step alternating kernel model and the random model and made numerous simulations for the dependence of $$\lambda _{1, \mathrm{twostep}}$$ and $$\varLambda $$ on the variance of the flow velocity $$v $$. In all our simulations, $$\lambda _{1, \mathrm{twostep}}$$ and $$\varLambda $$ are increasing functions of the variance of $$v$$. While we are not able to theoretically prove this result for these two models, Figs. 4, 5, and 9, 10, 11 were typical numerical examples chosen to illustrate the calculations.

Although our model, with uncorrelated random environments, showed that increasing temporal variations can promote population persistence, this phenomenon may not hold in other settings. For example, it has been shown that for a given average population growth rate, temporal variations in the growth rate may increase the risk of extinction; see e.g., Lewontin and Cohen (1969), Turelli (1978), Lande (1993), Halley and Iwasa (1999). Positive temporal autocorrelations in environmental conditions can decrease or increase extinction risk depending on other features; see, e.g., Schwager et al. (2006), Heino et al. (2000), Ripa and Lundberg (1996). In particular, positive autocorrelations in temporal fluctuations can disrupt predator-prey coexistence (Roth and Schreiber 2014). In more general and realistic situations where there are environmental variations in space and time, the effect of interactions between temporal correlations, spatial heterogeneity and dispersal on population persistence becomes even more complex. For instance, metapopulations whose expected fitness in every patch is less than $1$ can persist if there are positive temporal autocorrelations in relative fitness, sufficiently weak spatial correlations, and intermediate rates of dispersal between patches (Schreiber 2010). More recently, Roth and Schreiber (2014) develop a coexistence criterion for interacting structured populations in stochastic environments and show, among other applications, that autocorrelations in temporal fluctuations can interfere with coexistence in predator-prey models.

There is much further work that could be done. In this paper, we did not specifically address the critical domain size problem, other than illustrate how our method can be applied for an example with alternating kernels (Example 2). It is not our purpose here to study how the critical domain size is influenced by the variation of different factors, but this could be an interesting avenue for future work, especially for the random model, which would build upon the work for integrodifference equations in Kot and Schaffer (1986) for symmetric dispersal kernels and Hardin et al. (1988a, b, 1990), Van Kirk and Lewis (1997, 1999), Latore et al. (1999) for more general dispersal kernels, including environmental heterogeneity both in space and in time.

## Figures and Tables

**Fig. 13:**
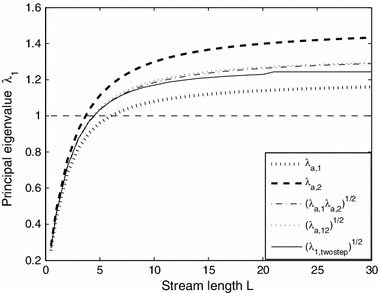
Comparison of principal eigenvalues with dispersal success approximations for the single and two-stage model as a function of stream length $L$. Parameter values are: $R_1 = 1.2$, $R_2 = 1.5$, $D_1 = D_2 = 1$, $\beta _1 = \beta _2 = 1$, $v_1 = 0.1$, and $v_2 = 1$

## Appendix

### A.1 Explicit solution for the principal eigenvalue for the alternating kernel model (27) in the symmetric case

Consider the case of the alternating kernel model (27) where

$$\begin{aligned} \displaystyle K_1(x)= \frac{a_1}{2} e^{-a_1|x|} \qquad \text { and } \qquad \displaystyle K_2(x) = \frac{a_2}{2} e^{-a_2|x|} \end{aligned}$$

are symmetric kernels with $a_1 =\sqrt{\beta _1/D_1}$ and $a_2 = \sqrt{\beta _2/D_2}$. In this case, the boundary-value problem (34) becomes

57 $$\begin{aligned} \phi ^{(4)}(x) - a \, \phi ^{(2)}(x) + d \, \phi (x)= 0, \end{aligned}$$

where

58 $$\begin{aligned} a = \left( a_1^2 + a_2^2 \right) \qquad \text { and } \qquad d = a_1^2 a_2^2 \left( 1 - \frac{\ R_1 R_2}{\lambda } \right) \end{aligned}$$

and the boundary conditions are

59 $$\begin{aligned} \phi '\left( 0 \right)&= a_2 \, \phi \left( 0 \right) \end{aligned}$$

60 $$\begin{aligned} \phi '\left( L \right)&= -a_2 \, \phi \left( L \right) \end{aligned}$$

61 $$\begin{aligned} \phi '''\left( 0 \right)&= a_1 \phi '' \left( 0 \right) + a_2^2 \left( a_2 - a_1\right) \phi \left( 0 \right) \end{aligned}$$

62 $$\begin{aligned} \phi '''\left( L \right)&= -a_1 \phi '' \left( L \right) - a_2^2 \left( a_2 - a_1 \right) \phi \left( L \right) \!. \end{aligned}$$

The characteristic equation for (57) is $r^4 - a r^2 + d = 0$ with roots

63 $$\begin{aligned} \left\{ \pm \sqrt{\frac{a + \sqrt{a^2 - 4d}}{2}}, \pm \sqrt{\frac{a - \sqrt{a^2 - 4d}}{2}} \right\} . \end{aligned}$$

The first two roots are real and since $d < 0$ (by estimate (28)), the second two roots are complex (and purely imaginary). If we denote the real roots by $\pm r$ and the complex roots by $\pm i k$ then the general solution of (57) is

$$\begin{aligned} y(x) = c_1 \cosh r x + c_2 \sinh r x + c_3 \cos k x + c_4 \sin k x. \end{aligned}$$

By shifting the domain from $[0,L]$ to $[-L/2,L/2]$ we can use symmetry to reduce this to

$$\begin{aligned} y(x) = c_1 \cosh r x + c_3 \cos k x. \end{aligned}$$

Applying the boundary conditions (59) and (61) (which now hold at $-L/2$) we obtain the $2 \times 2$ system of equations for $c_1$ and $c_3$:

$$\begin{aligned}&\left( a_2 \, \cosh \frac{rL}{2} + r \, \sinh \frac{rL}{2} \right) c_1 + \left( a_2 \, \cos \frac{kL}{2} - k \, \sin \frac{kL}{2} \right) c_3 = 0,\\&\quad \left( (a_1 r^2 + a_2^2 (a_2 - a_1)) \cosh \frac{rL}{2} + r^3 \sinh \frac{rL}{2} \right) c_1\\&\quad + \left( k^3 \sin \frac{kL}{2} - (a_1 k^2 - a_2^2 (a_2 - a_1) )\cos \frac{kL}{2} \right) c_3 = 0. \end{aligned}$$

This system admits a nontrivial solution only if the determinant of the coefficient matrix is zero, which yields an equation of the form

64 $$\begin{aligned} F(L, a_1, a_2, R_1 R_2,\lambda ) = 0 \end{aligned}$$

which implicitly defines the principal eigenvalue $\lambda _{1,\mathrm{twostep}}$. Furthermore, by setting $\lambda =1$ in (64), we obtain an implicit equation for the critical domain length $L = L(a_1, a_2, R_1 R_2)$.

### A.2 Redistribution and dispersal success approximations for alternating kernel model

Suppose $\phi _1$ is an eigenfunction associated with the alternating kernel model, i.e., $\phi _1$ solves

65 $$\begin{aligned} \lambda _{1,\mathrm{twostep}} \, \phi _1(x) = R_1 R_2 \int _{\varOmega } K(x,y) \, \phi _1(y) \, dy \end{aligned}$$

where $K$ is the two-stage kernel (26). Integrating (65) over $\varOmega$ yields

$$\begin{aligned} \lambda _{1,\mathrm{twostep}} \int _{\varOmega } \phi _1(x) \, dx&= R_1 R_2 \int _{\varOmega } \int _{\varOmega } K(x,y) \phi _1(y) \, dy \, dx \\&= R_1 R_2 \int _{\varOmega } \left[ ~ \int _{\varOmega } K(x,y) \, dx \right] \phi _1(y) \, dy \\&= R_1 R_2 \int _{\varOmega } s(y) \, \phi _1(y) \, dy, \end{aligned}$$

where $s(y)$ is the dispersal success function for $K$. If $\varOmega = [0,L]$ and we use the approximation $\phi _1 \approx 1/L$ we obtain

$$\begin{aligned} \lambda _{1,\mathrm{twostep}} \approx \frac{R_1 R_2}{L} \int _{\varOmega } s(y) \, dy&= \frac{R_1 R_2}{L} \int _{\varOmega } \int _{\varOmega } \int _{\varOmega } K_2(x,z) K_1(z,y) \, dz \, dx \, dy \\&= \frac{R_1 R_2}{L} \int _{\varOmega } \int _{\varOmega } s_2(z) K_1(z,y) \, dz \, dy \\&= \frac{R_1 R_2}{L} \int _{\varOmega } s_2(z) \, r_1(z) \, dz. \end{aligned}$$

Therefore, we obtain the approximation

66 $$\begin{aligned} \lambda _{1,\mathrm{twostep}} \approx \lambda _{a,12} := \frac{R_1 R_2}{L} \int _{\varOmega } s_2(y) \, r_1(y) \, dy \end{aligned}$$

which approximates $\lambda _{1,\mathrm{twostep}}$ in terms of the dispersal success function (9) for $K_2$ and redistribution function (10) for $K_1$.

To compare the estimates for the alternating kernel model we consider (27) on $\varOmega =[0,L]$ with kernels $K_1$ and $K_2$ defined by (5). We denote the dispersal success approximations for the single stage case with either $K_1$ or $K_2$ as

67 $$\begin{aligned} \lambda _{1}(K_1) \approx \lambda _{a,1}&:= \frac{R_1}{L} \int _{\varOmega } s_1(y) \, dy, \end{aligned}$$

68 $$\begin{aligned} \lambda _{1}(K_2) \approx \lambda _{a,2}&:= \frac{R_2}{L} \int _{\varOmega } s_2(y) \, dy. \end{aligned}$$

Figure 13 shows an example of the comparison of the approximations for the principal eigenvalues of the single stage and two stage operators with $\lambda _{1,\mathrm{twostep}}$, the actual principal eigenvalue, obtained by solving the boundary value problem (34)–(40). The principal eigenvalues $\lambda _{1,\mathrm{twostep}}$ and $(\lambda _{1,\mathrm{twostep}})^{1/2}$ both increase as $L$ increases (as one would expect), $\lambda _{a,1}$ underestimates $\lambda _{1,\mathrm{twostep}}$ but $\lambda _{a,2}$ overestimates $\lambda _{1,\mathrm{twostep}}$. The geometric mean $(\lambda _{a,1}\lambda _{a,2})^{1/2}$ of one year approximations appears to provide a good estimate for $(\lambda _{1,\mathrm{twostep}})^{1/2}$ when $L$ is not too large, but it overestimates $(\lambda _{1,\mathrm{twostep}})^{1/2}$ when $L$ is large. The geometric mean $(\lambda _{a,1}\lambda _{a,2})^{1/2}$ also tends to underestimate the two-step approximation $(\lambda _{a,12})^{1/2}$, which itself overestimates the actual persistence measure $(\lambda _{1,\mathrm{twostep}})^{1/2}$. One should note that this conclusion is based on the results for the single model and the two-stage model with chosen parameters in the example.

### A.3 Proof of Theorem 1

First we recall some preliminary facts from Hardin et al. (1988a, 1990). Consider the general model:

69 $$\begin{aligned} x_{t+1}(\omega )=F_{\alpha _t}(x_t(\omega )), \end{aligned}$$

where $t=0,1,2,\ldots$, $\omega \in \varOmega$, $\varOmega$ is a compact set in ${\mathbb {R}}^n$ for some $n\ge 1$, and $x_0 \in C_+(\varOmega )$. They consider the following assumptions:

(C0) $\alpha _t$ is a sequence of independent identically distributed random variables in some index set $\mathcal {A}$.
(H1) For each $\alpha \in \mathcal {A}$, $F_\alpha$ is a continuous map of $C_+(\varOmega )$ into itself such that $F_\alpha (x)=0\in C_+(\varOmega )$ if and only if $x=0\in C_+(\varOmega )$.
(H2) If $x, y\in C_+(\varOmega )$ and $x\ge y$ then $F_\alpha (x)\ge F_\alpha (y)$.
(H3) There exists $b>0$ such that for $x\in C_+(\varOmega )$
  - $\Vert F_\alpha (x)\Vert _\infty \le b$ for all $\alpha \in \mathcal {A}$ whenever $\Vert x\Vert _\infty \le b$;
  - there exists $t$ (depending on $x \in C_+(\varOmega )$) such that $\Vert F_{\alpha _t}\circ \cdots \circ F_{\alpha _0} \, x \Vert _\infty <b$ for all $\alpha _0, \ldots ,\alpha _t\in \mathcal {A}$;
  - there exists $$d>0 $$ such that $$F_\alpha (b)\ge d $$ for all $$\alpha \in \mathcal {A}$$.
(H4) Let $B_b=\{x\in C_+(\varOmega ):\Vert x\Vert _\infty \le b\}$ where $b$ is as in (H3). Then there is some compact set $D\subset C_+(\varOmega )$ such that $F_\alpha (B_b)\subset D$ for all $\alpha \in \mathcal {A}$.
(H5) There exists $h>0$ such that $\Vert F_\alpha (x)\Vert _\infty \le h\Vert x\Vert _\infty$ for all $\alpha \in \mathcal {A}$ and $x\in C_+(\varOmega )$.
(H6) There exists $\xi >0$ such that $F_\alpha (B_b)\subset K_\xi$ where $K_\xi =\{x\in C_+(\varOmega ):x\ge \xi \Vert x\Vert _\infty \}$.
(H7) For each $a>0$ there exists a continuous function $\tau :(0,1]\rightarrow (0,1]$ such that $\tau (s)>s$ for $s\in (0,1)$ and $\tau (s)F_\alpha (x)\le F_\alpha (sx)$ for all $\alpha \in \mathcal {A}$ and $x\in C_+(\varOmega )$ for which $a \le x\le b$.
(H8) $F_\alpha$ is Fréchet differentiable (with respect to $C_+(\varOmega ))$ at $0\in C_+(\varOmega )$. We write $\fancyscript{L}_\alpha$ for the derivative $F_\alpha ^\prime (0)$.
(H9) There exists a function $N:\mathbb {R}_+\rightarrow [0,1]$ such that $\lim \limits _{u\rightarrow 0^+} N(u)=1$ and $N(\Vert x\Vert _\infty )\fancyscript{L}_\alpha x\le F_\alpha (x)\le \fancyscript{L}_\alpha x$ for $x\in C_+(\varOmega )$.
  The above assumptions imply the following additional conditions:
(H10) - If $x, y\in C(\varOmega )$ and $x\le y$ then $\fancyscript{L}_\alpha x\le \fancyscript{L}_\alpha y$ for all $\alpha \in \mathcal {A}$.
  - $$\Vert \fancyscript{L}_\alpha \Vert =\sup \{\Vert \fancyscript{L}_\alpha x\Vert _\infty :x\in C(\varOmega ), \Vert x\Vert _\infty =1\}=\Vert \fancyscript{L}_\alpha (1)\Vert _{\infty }$$ for all $$\alpha \in \mathcal {A}$$.
  - $$\Vert \fancyscript{L}_\alpha \Vert \le h $$ for all $$\alpha \in \mathcal {A}$$.
  - $$\fancyscript{L}_\alpha (C_+(\varOmega ))\subset K_\xi $$ for all $$\alpha \in \mathcal {A}$$.
  (H11) There exists $\gamma >0$ such that if $x\in K_\xi$ and $\Vert x\Vert _\infty \le b$ then $F_\alpha (x)\ge \gamma \Vert x\Vert _\infty$ for all $\alpha \in \mathcal {A}$.

Under these assumptions Hardin et al. (1988a) prove the following theorems for persistence:

#### **Theorem 4.2**

Suppose the model (69) satisfies (C0), (H1)–(H7) and that $x_0\ne 0$, $x_0\in C_+(\varOmega )$ with probability one. Then $x_t$ converges in distribution to a stationary distribution $\mu ^*$, independent of $x_0$, such that either $\mu ^*(\{0\})=0$ or $\mu ^*(\{0\})=1$.

#### **Lemma 5.1**

Suppose $\{\fancyscript{L}_{\alpha _t}\}$ satisfies (C0) and (H10)(c). Then $\lim \nolimits _{t\rightarrow \infty } \Vert \fancyscript{L}_{\alpha _t}\circ \cdots \circ \fancyscript{L}_{\alpha _1}\Vert ^{1/t}$ exists with probability one and is a constant $r$ (independent of the initial state).

#### **Theorem 5.3**

Suppose $F_\alpha$ satisfies (C0), (H1)–(H9) and that $x_0\ne 0, x_0\in C_+(\varOmega )$ with probability one. Let $\mu ^*$ be as in Theorem 4.2.

- If $r<1$, then $\mu ^*(\{0\})=1$ and $x_t\rightarrow 0$ with probability one.
- If $$r>1$$, then $$\mu ^*(\{0\})=0$$.

Therefore, in order to prove Theorem 1 we first verify that

$$\begin{aligned} F_{\alpha }(n)(x) =\int _\varOmega K_{\alpha }(x-y)f_{\alpha }(n(y))\,dy \end{aligned}$$

(as in (44)) with the hypotheses (C1)–(C3) satisfies (H1)–(H9). For notational simplicity we write $|\varOmega |=\int _\varOmega \,dy$.

1. For any $\alpha \in \mathcal {A}$, the continuity of $F_{\alpha }$ in $C_+(\varOmega )$ follows from the continuity assumption on $K_{\alpha }$ and $f_{\alpha }$. Moreover, (C1) and (C2) imply that $F_{\alpha }(u)=0$ if and only if $u=0$.
2. By the monotonicity of $f_{\alpha }$, for any $\alpha \in \mathcal {A}$ and $u\ge v$ in $C_+(\varOmega )$ we have
  $$\begin{aligned} F_{\alpha }(u)=\int _\varOmega K_{\alpha }(x-y)f_{\alpha }(u(y))\,dy\ge \int _\varOmega K_{\alpha }(x-y)f_{\alpha }(v(y))\,dy=F_{\alpha }(v). \end{aligned}$$
3. - Recall by (C2)(b), that $f_{\alpha }(u) \le m$. Let $b=m\overline{K}|\varOmega |$. Then for any $\alpha \in \mathcal {A}$ and $u\in C_+(\varOmega )$ with $\Vert u\Vert _\infty \le b$, we have
    $$\begin{aligned} \Vert F_{\alpha }(u)\Vert _\infty&= \max \limits _{x\in \varOmega }\int _\varOmega K_{\alpha }(x-y)f_{\alpha }(u(y))\,dy \\&\le \max \limits _{x\in \varOmega }\int _\varOmega K_{\alpha }(x-y) f_{\alpha }(b)\,dy \le m \, \overline{K} |\varOmega | = b. \end{aligned}$$
  - Similar to (a), the bound $f_{\alpha }(u) \le m$ implies $\Vert F_{\alpha }(u)\Vert _{\infty } \le b$ for all $\alpha \in \mathcal {A}$ and $u \in C_+(\varOmega )$, hence (H3)(b) holds for all $t > 0$.
  - Let $d=\underline{f_1} \underline{K} |\varOmega |$. Then $d>0$ and for any $\alpha \in \mathcal {A}$,
    $$\begin{aligned} F_{\alpha }(b)=\int _\varOmega K_{\alpha }(x-y) \, f_{\alpha }(b)\, dy \ge \underline{f_1} \, \underline{K} \, |\varOmega |=d. \end{aligned}$$
4. First we show that $F_{\alpha }$ is compact for each $\alpha \in \mathcal {A}$. Let $\{u_k\}$ be a bounded sequence in $C(\varOmega )$. Then for any $\alpha \in \mathcal {A}$ we have
  $$\begin{aligned} \Vert F_{\alpha }(u_k)\Vert _\infty =\max \limits _{x\in \varOmega } \left| \int _\varOmega K_{\alpha }(x-y)f_{\alpha }(u_k(y)) \, dy \right| \le m \overline{K}|\varOmega |=b \end{aligned}$$
  which shows $\{F_{\alpha }(u_k)\}$ is uniformly bounded. Next, for any $x_1,x_2 \in \varOmega$ we have
  $$\begin{aligned} \left| F_{\alpha }(u_k)(x_1)-F_{\alpha }(u_k)(x_2) \right|&= \left| \int _\varOmega [K_{\alpha }(x_1-y)-K_{\alpha }(x_2-y)]f_{\alpha }(u_k(y))\,dy \right| \\&\le m \int _\varOmega |K_{\alpha }(x_1-y)-K_{\alpha }(x_2-y)|\,dy. \end{aligned}$$
  Since $K_{\alpha }$ is continuous, it follows $\{F_{\alpha }(u_k)\}$ is equicontinuous. Thus, by the Arzelà–Ascoli theorem, $\{F_{\alpha }(u_k)\}$ has a convergent subsequence which implies $F_{\alpha }$ is a compact map. Therefore, for any $\alpha \in \mathcal {A}$, $\overline{F_\alpha (B_b)}$ is compact. By assumption $(C3)$, $\overline{F_\alpha (B_b)}\subset \overline{F_{\alpha ^*}(B_b)}$ for all $\alpha \in {\mathcal {A}}$. Let $D=\overline{F_{\alpha ^*}(B_b)}$. Then we have $F_{\alpha }(B_b) \subset D$ for all $\alpha \in {\mathcal {A}}$. Therefore, (H4) is true.
5. Since $f_{\alpha }(u)\le f_{\alpha }^\prime (0)u$ for all $u\ge 0$ and $f_{\alpha }^\prime (0)\in [\underline{f},\overline{f}]$, it follows that
  $$\begin{aligned} \Vert F_{\alpha }(u)\Vert _\infty \le \max \limits _{x\in \varOmega } \int _\varOmega K_{\alpha }(x-y) \, f_{\alpha }^\prime (0)\, u(y)\, dy \le h \Vert u\Vert _{\infty }, \end{aligned}$$
  where $$h=\overline{f} \, \overline{K} \, |\varOmega |$$.
6. For any $u\in B_b$ we have $F_{\alpha }(u)\in B_b\subset C_+(\varOmega )$. We want $\xi >0$ such that $F_{\alpha }(u)(x)\ge \xi \Vert F_{\alpha }(u)\Vert _\infty$ for all $x\in \varOmega$. Similar to the estimate in (H5), for any $x\in \varOmega$ we have
  $$\begin{aligned} F_{\alpha }(u)(x) \le \overline{f} \, \overline{K}\int _\varOmega u(y)\,dy. \end{aligned}$$
  Thus $\Vert F_{\alpha }(u)\Vert _\infty \le \overline{f} \, \overline{K}\int _\varOmega u(y)\,dy$, and hence
  $$\begin{aligned} \int _\varOmega u(y)dy\ge \frac{\Vert F_{\alpha }(u)\Vert _\infty }{\overline{f}\overline{K}}. \end{aligned}$$
  On the other hand, by (C2), for any $u\in B_b$ and $y\in \varOmega$ we have $\frac{f_{\alpha }(b)}{b} \le \frac{f_{\alpha }(u(y))}{u(y)}$, and hence
  $$\begin{aligned} F_{\alpha }(u)(x)&= \int _\varOmega K_{\alpha }(x-y)f_{\alpha }(u(y))\,dy \\&\ge \int _\varOmega K_{\alpha }(x-y) \, \frac{f_{\alpha }(b)}{b} \, u(y) \, dy\\&\ge \frac{\underline{f_1} \, \underline{K}}{b}\int _\varOmega u(y)\,dy \\&\ge \frac{\underline{f_1}\underline{K}}{b} \ \frac{\Vert F_{\alpha }(u)\Vert _\infty }{\overline{f} \, \overline{K}} = \frac{\underline{f_1}}{b \overline{f}} \frac{\underline{K}}{\overline{K}} \, \Vert F_{\alpha }(u)\Vert _\infty . \end{aligned}$$
  This implies (H6) holds with $\xi =\frac{\underline{f_1}}{b \overline{f}}\frac{\underline{K}}{\overline{K}}$. Moreover, $\xi \in (0,1)$ by the choice of $\underline{f_1}$, $b$, $\overline{f}$, $\underline{K}$ and $\overline{K}$.
7. We show that there exists $\tau (s)$ such that $f_\alpha (su)\ge \tau (s)f_\alpha (u)$. To this end, let $a>0$ with $a\le b$. By (C2), for any $s\in (0,1)$, $u>0$ and $\alpha \in \mathcal {A}$, we have $f_{\alpha }(su)/f_{\alpha }(u)>s$. Define $\tau :(0,1]\rightarrow (0,1]$ by $\tau (s)=\min \nolimits _{\alpha \in \mathcal {A},a\le u\le b}\frac{f_{\alpha }(su)}{f_{\alpha }(u)}$. Then $\tau (s)>s$, for all $s\in (0,1)$ and $\frac{f_{\alpha }(su)}{f_{\alpha }(u)}\ge \tau (s)$ for any $\alpha \in \mathcal {A}$, $s\in (0,1)$, $a\le u\le b$. Then for any $s\in (0,1)$ and $a \le u\le b$,
  $$\begin{aligned} F_{\alpha }(su)(x) =\int _\varOmega K_{\alpha }(x-y) f_{\alpha }(su(y))\,dy&\ge \int _\varOmega K_{\alpha }(x-y)\tau (s)f_{\alpha }(u(y))\,dy\\&= \tau (s)\int _\varOmega K_{\alpha }(x-y)f_{\alpha }(u(y))\,dy\\&= \tau (s)F_{\alpha }(u)(x). \end{aligned}$$
8. The Fréchet derivative of $F_{\alpha }$ (in $C_+(\varOmega )$) is the linear operator on $C_+(\varOmega )$ given by
  $$\begin{aligned} \fancyscript{L}_\alpha (u):=F_{\alpha }^\prime (0)(u)(x)=\int _\varOmega K_{\alpha }(x-y)f_{\alpha }^\prime (0)u(y)\,dy. \end{aligned}$$
9. By (C2), $f_{\alpha }(u)\le f_{\alpha }^\prime (0)u$ for all $u\in C_+(\varOmega )$ and $\alpha \in \mathcal {A}$. Thus
  $$\begin{aligned} F_{\alpha }(u)=\int _\varOmega K_{\alpha }(x-y) f_{\alpha }(u(y))\,dy\le \int _\varOmega K_{\alpha }(x-y) f_{\alpha }^\prime (0)u(y)\,dy =\fancyscript{L}_\alpha (u) \end{aligned}$$
  for all $u\in C_+(\varOmega )$. For each $\alpha \in {\mathcal {A}}$, define $N_\alpha :[0,\infty ) \rightarrow [0,1]$ by
  $$\begin{aligned} N_\alpha (u)={\left\{ \begin{array}{ll} \displaystyle \frac{f_{\alpha }(u)}{f_{\alpha }^\prime (0)u}, &{} \text { if } u>0,\\ 1, &{} \text { if } u=0. \end{array}\right. } \end{aligned}$$
  Then $\lim \nolimits _{u\rightarrow 0^+}N_\alpha (u)=1$ for any $\alpha \in \mathcal {A}$. Let $N(u)=\min \nolimits _{\alpha \in \mathcal {A}}N_\alpha (u)$. It follows from the limit of $N_\alpha$ and choice of $N(u)$ that $\lim \nolimits _{u\rightarrow 0^+}N(u)=1$. By (C2), we have
  $$\begin{aligned} \frac{f_{\alpha }(u(x))}{u(x)}\ge \frac{f_{\alpha }(\Vert u\Vert _\infty )}{\Vert u\Vert _\infty } \end{aligned}$$
  and hence,
  $$\begin{aligned} \frac{f_{\alpha }(u(x))}{u(x)f_{\alpha }^\prime (0)}\ge \frac{f_{\alpha }(\Vert u\Vert _\infty )}{\Vert u\Vert _\infty f_{\alpha }^\prime (0)}, \end{aligned}$$
  i.e., $$N_\alpha (u(x))\ge N_\alpha (\Vert u\Vert _\infty )$$. Moreover,
  $$\begin{aligned} F_{\alpha }(u)(x) = \int _\varOmega K_{\alpha }(x-y)f_{\alpha }(u(y))\,dy&= \int _\varOmega K_{\alpha }(x-y)f_{\alpha }^\prime (0)u(y)N_\alpha (u(y))\,dy\\&\ge \int _\varOmega K_{\alpha }(x-y)f_{\alpha }^\prime (0)u(y)N_\alpha (\Vert u\Vert _\infty )\,dy\\&= N_\alpha (\Vert u\Vert _\infty )\fancyscript{L}_\alpha (u)(x) \\&\ge N(\Vert u\Vert _\infty )\fancyscript{L}_\alpha (u)(x). \end{aligned}$$
  Items (H10) and (H11) follow from the earlier properties. For the sake of clarity, we include the additional proofs.
10. - For any $u,v \in C(\varOmega )$ with $u\le v$ and $x\in \varOmega$,
    $$\begin{aligned} \fancyscript{L}_\alpha (u)(x)&= \int _\varOmega K_\alpha (x-y)f_\alpha ^\prime (0)u(y)\,dy\le \int _\varOmega K_\alpha (x-y)f_\alpha ^\prime (0)v(y)\,dy\\&= \fancyscript{L}_\alpha (v)(x). \end{aligned}$$
  - If $\Vert u \Vert _{\infty } \le 1$ then $u(x) \le 1$ for all $x$, and it follows from monotonicity of $\fancyscript{L}_\alpha$ that $\fancyscript{L}_\alpha (u) \le \fancyscript{L}_\alpha (1)$, hence $\Vert \fancyscript{L}_\alpha (u) \Vert _{\infty } \le \Vert \fancyscript{L}_\alpha (1) \Vert _{\infty }$ and so $\displaystyle \Vert \fancyscript{L}_\alpha \Vert =\sup \nolimits _{\Vert u \Vert _{\infty } = 1} \Vert \fancyscript{L}_\alpha (u) \Vert _\infty = \Vert \fancyscript{L}_{\alpha } (1) \Vert _{\infty }$ for all $\alpha \in \mathcal {A}$.
  - By a similar calculation as in (H5), it follows
    $$\begin{aligned} \Vert \fancyscript{L}_\alpha u\Vert _\infty \le h \Vert u\Vert _\infty \end{aligned}$$
    for all $\alpha \in \mathcal {A}$ and $u\in C(\varOmega )$, which implies that $\Vert \fancyscript{L}_\alpha \Vert \le h = \overline{f} \, \overline{K} |\varOmega |$ for all $\alpha \in \mathcal {A}$.
  - Let $u\in C_+(\varOmega )$ and $\alpha \in \mathcal {A}$. For any $x\in \varOmega$, we have
    $$\begin{aligned}&\Vert \fancyscript{L}_\alpha (u)\Vert _\infty \le \overline{f} \, \overline{K} \int _\varOmega u(y)\,dy \quad \text { and hence }\\&\quad \int _\varOmega u(y)\,dy\ge \frac{\Vert \fancyscript{L}_\alpha (u)\Vert _\infty }{\overline{f} \, \overline{K}}. \end{aligned}$$
    Thus for any $x\in \varOmega$,
    $$\begin{aligned} \fancyscript{L}_\alpha (u)(x)\!=\!\int _\varOmega K_\alpha (x\!-\!y)f_\alpha ^\prime (0)u(y)\,dy \!\ge \! \underline{f} \underline{K}\int _\varOmega \! u(y)\,dy \!\ge \! \frac{\underline{f} \underline{K}}{\overline{f} \, \overline{K}}\Vert \fancyscript{L}_\alpha (u)\Vert _\infty . \end{aligned}$$
    Since $\underline{f_1}=\inf \nolimits _{\alpha \in {\mathcal {A}}}f_\alpha (b)\le \inf \nolimits _{\alpha \in {\mathcal {A}}}f_\alpha ^\prime (0)b=\underline{f}b$, we have $\underline{f}\ge \underline{f_1}/b$, and hence, for any $x\in \varOmega$,
    $$\begin{aligned} \fancyscript{L}_\alpha (u)(x)\ge \frac{\underline{f} \underline{K}}{\overline{f} \, \overline{K}}\Vert \fancyscript{L}_\alpha (u)\Vert _\infty \ge \frac{\underline{f_1} \underline{K}}{b\overline{f} \, \overline{K}}\Vert \fancyscript{L}_\alpha (u)\Vert _\infty =\xi \, \Vert \fancyscript{L}_\alpha (u)\Vert _\infty . \end{aligned}$$
11. Assume that $u \in K_\xi$ with $\Vert u\Vert _\infty \le b$. Then $u(x) \ge \xi \Vert u\Vert _\infty$ and for any $\alpha \in \mathcal {A}$ we have
  $$\begin{aligned} F_\alpha (u)\ge F_\alpha (\xi \, \Vert u\Vert _\infty )=F_\alpha \left( \frac{\xi \Vert u\Vert _\infty }{b} \, b\right) . \end{aligned}$$
  Since $\frac{\xi \Vert u\Vert _\infty }{b}\le 1$, it follows from (H3) and (H7) that
  $$\begin{aligned} F_{\alpha }(u) \ge F_\alpha \left( \frac{\xi \Vert u\Vert _\infty }{b}b\right) \ge \frac{\xi \Vert u\Vert _\infty }{b}F_\alpha (b)\ge \frac{\xi \Vert u\Vert _\infty }{b} d=\gamma \Vert u\Vert _\infty \end{aligned}$$
  where $$\gamma =\xi d/b$$.

By (H1)–(H9) it follows from Theorem 4.2 in Hardin et al. (1988a) that for all $n_0\ne 0$, $n_0\in C_+(\varOmega )$ with probability one, $n_t$ converges in distribution to a stationary distribution $\mu ^*$, independent of $n_0$, such that either $\mu ^*(\{0\})=0$ or $\mu ^*(\{0\})=1$. By (H10)(c) we know $\Vert \fancyscript{L}_\alpha \Vert _\infty \le h$ for all $\alpha \in {\mathcal {A}}$. Thus from Lemma 5.1 in Hardin et al. (1988a) it follows that $\lim \nolimits _{t\rightarrow \infty } \Vert \fancyscript{L}_{\alpha _t} \circ \cdots \circ \fancyscript{L}_{\alpha _1}\Vert ^{1/t}$ exists with probability one, and hence we can define a constant $r$ as

$$\begin{aligned} r=\lim \limits _{t\rightarrow \infty } \Vert \fancyscript{L}_{\alpha _t}\circ \cdots \circ \fancyscript{L}_{\alpha _1}\Vert ^{1/t}. \end{aligned}$$

Finally, it follows from Theorem 5.3 in Hardin et al. (1988a) that if $r<1$, then $\mu ^*(\{0\})=1$ and $n_t\rightarrow 0$ with probability one and if $r>1$, then $\mu ^*(\{0\})=0$. In this sense, if $r<1$ then, in the long run, the population density approaches zero with probability one (extinction), while if $r>1$ the population density will not approach zero, and hence persists.

### A.4 Proof of Theorem 2

First, we consider the case of constant initial data.

Let $n_t(x)$ be the solution of (46) with initial function $n_0(x) = C > 0$. Note that

$$\begin{aligned} \Vert n_t\Vert _\infty =\sup \limits _{x\in \varOmega } n_t(x)=\sup \limits _{x\in \varOmega }\fancyscript{L}_{\alpha _t} \circ \cdots \circ \fancyscript{L}_{\alpha _1} n_0(x)\le \Vert \fancyscript{L}_{\alpha _t} \circ \cdots \circ \fancyscript{L}_{\alpha _1}\Vert \, \Vert n_0\Vert _\infty . \end{aligned}$$

Thus,

$$\begin{aligned} \left( ~\int _\varOmega n_t(x)dx\right) ^{1/t}\le (\Vert n_t\Vert _\infty \, |\varOmega |)^{1/t}\le \Vert \fancyscript{L}_{\alpha _t} \circ \cdots \circ \fancyscript{L}_{\alpha _1}\Vert ^{1/t}\, (\Vert n_0\Vert _\infty \, |\varOmega |)^{1/t}. \end{aligned}$$

Since $\lim \nolimits _{t\rightarrow \infty } (\Vert n_0\Vert _\infty \, |\varOmega |)^{1/t}=1$, the above inequality implies

$$\begin{aligned} \limsup \limits _{t\rightarrow \infty }\left( ~\int _\varOmega n_t(x)dx\right) ^{1/t}&\le \limsup \limits _{t\rightarrow \infty }\Vert \fancyscript{L}_{\alpha _t} \circ \cdots \circ \fancyscript{L}_{\alpha _1}\Vert ^{1/t}\, (\Vert n_0\Vert _\infty \, |\varOmega |)^{1/t} \\&= \lim \limits _{t\rightarrow \infty }\Vert \fancyscript{L}_{\alpha _t} \circ \cdots \circ \fancyscript{L}_{\alpha _1}\Vert ^{1/t} = r. \end{aligned}$$

On the other hand, (H10)(b) implies $\displaystyle \Vert \fancyscript{L}_\alpha \Vert = \Vert \fancyscript{L}_{\alpha } (1) \Vert _{\infty }$ for all $\alpha \in \mathcal {A}$. By a similar argument as in the proof of (H10)(b), it follows

$$\begin{aligned} \Vert \fancyscript{L}_{\alpha _t} \circ \cdots \circ \fancyscript{L}_{\alpha _1}\Vert =\Vert \fancyscript{L}_{\alpha _t} \circ \cdots \circ \fancyscript{L}_{\alpha _1}(1)\Vert _\infty \end{aligned}$$

for all $$\alpha _1,\ldots , \alpha _t\in \mathcal {A}$$.

Therefore,

$$\begin{aligned} r=\lim \limits _{t\rightarrow \infty } \Vert \fancyscript{L}_{\alpha _t}\circ \cdots \circ \fancyscript{L}_{\alpha _1}\Vert ^{1/t}=\lim \limits _{t\rightarrow \infty }\Vert \fancyscript{L}_{\alpha _t} \circ \cdots \circ \fancyscript{L}_{\alpha _1}(1)\Vert _\infty ^{1/t}. \end{aligned}$$

Consider the sequence $\{n_t(x)\}_{t\in \mathbb {N}}$. By (H10)(d) there exists $\xi \in (0,1)$ such that $\fancyscript{L}_{\alpha _t}(C_+(\varOmega ))\subset K_\xi$ for all $\alpha _t\in {\mathcal {A}}$, where $K_\xi =\{u\in C_+(\varOmega ):u(x)\ge \xi \Vert u\Vert _\infty , \text { for all } x\in \varOmega \}$ (in terms of the kernels and growth functions, $\xi =\underline{f_1}\underline{K}/(b\overline{f} \, \overline{K})$, as shown in (H6)). In particular, for each $t \in \mathbb {N}$, $n_t(x)\ge \xi \Vert n_t\Vert _\infty$. Note that

$$\begin{aligned} n_t(x)=\fancyscript{L}_{\alpha _t} \circ \cdots \circ \fancyscript{L}_{\alpha _1}(C)=C\cdot \fancyscript{L}_{\alpha _t} \circ \cdots \circ \fancyscript{L}_{\alpha _1}(1), \ \forall t\ge 0. \end{aligned}$$

Therefore,

$$\begin{aligned} \begin{array}{rl} \displaystyle \int _\varOmega n_t(x)dx \ge \xi \Vert n_t\Vert _\infty \, |\varOmega | &{} = \xi |\varOmega | C \cdot \Vert \fancyscript{L}_{\alpha _t} \circ \cdots \circ \fancyscript{L}_{\alpha _1}(1)\Vert _\infty \\ &{} = \xi |\varOmega | C \cdot \Vert \fancyscript{L}_{\alpha _t} \circ \cdots \circ \fancyscript{L}_{\alpha _1}\Vert . \end{array} \end{aligned}$$

Since $\lim \nolimits _{t\rightarrow \infty }(\xi \Vert \varOmega \Vert C)^{1/t}=1$, we have

$$\begin{aligned} \liminf \limits _{t\rightarrow \infty }\left( ~\int _\varOmega n_t(x)dx\right) ^{1/t}\ge \liminf \limits _{t\rightarrow \infty }(\xi |\varOmega | C)^{1/t}\cdot \Vert \fancyscript{L}_{\alpha _t} \circ \cdots \circ \fancyscript{L}_{\alpha _1}\Vert ^{1/t}=r. \end{aligned}$$

Therefore, for the solution $n_t(x)$ of (46) with the initial function $n_0(x)\equiv C > 0$, we have

$$\begin{aligned} r\le \liminf \limits _{t\rightarrow \infty }\left( \,\,\int _\varOmega n_t(x)\,dx\right) ^{1/t}\le \limsup \limits _{t\rightarrow \infty }\left( \,\,\int _\varOmega n_t(x)dx\right) ^{1/t}\le r, \end{aligned}$$

which implies that $\lim \nolimits _{t\rightarrow \infty }(~\int _\varOmega n_t(x)\,dx)^{1/t}$ exists and

$$\begin{aligned} \lim \limits _{t\rightarrow \infty }\left( ~\int _\varOmega n_t(x)\,dx\right) ^{1/t}=r. \end{aligned}$$

Now let $n_t(x)$ be a solution of (46) with initial function $n_0\in C_+(\varOmega )\backslash \{0\}$. If $n_0(x_0)=0$ for some $x_0\in \varOmega$, then by (H10)(d) we know $n_1(x) > 0$ in $\varOmega$ so, shifting by one if necessary, we can assume $n_0(x) > 0$ for all $x \in \varOmega$. Since $\varOmega$ is closed and bounded, there exist constants $\underline{m}, \, \overline{m}$ with $0< \underline{m} \le \overline{m}$ such that $\underline{m} \le n_0(x)\le \overline{m}$ for $x \in \varOmega$. Let $\underline{n}_t(x)$ and $\overline{n}_t(x)$ be solutions of (46) with initial functions $\underline{n}_0(x)\equiv \underline{m}$ and $\overline{n}_0(x)\equiv \overline{m}$, respectively. Then by the above result for constant initial data, we have

$$\begin{aligned} r\!=\!\lim \limits _{t\rightarrow \infty }\left( ~\int _\varOmega \underline{n}_t(x)\,dx\right) ^{1/t}\le \lim \limits _{t\rightarrow \infty }\left( ~\int _\varOmega n_t(x)\,dx\right) ^{1/t}\le \lim \limits _{t\rightarrow \infty }\left( ~\int _\varOmega \overline{n}_t (x)\,dx\right) ^{1/t}=r. \end{aligned}$$

Therefore, $$\lim \nolimits _{t\rightarrow \infty }(\int \nolimits _\varOmega n_t(x)\,dx)^{1/t}=r$$.

We have shown that $\lim \nolimits _{t\rightarrow \infty }(\int \nolimits _\varOmega n_t(x)\,dx)^{1/t}=r$ for any solution of (46) with initial value $n_0\in C_+(\varOmega )\backslash \{0\}$. Furthermore, if $n_0\in C_+(\varOmega )\backslash \{0\}$, then $\int \nolimits _{\varOmega } n_0(x) \, dx > 0$ and hence $\lim \nolimits _{t\rightarrow \infty }(\int \nolimits _\varOmega n_0(x)\,dx)^{1/t}=1$. Therefore, the limit in (47) exists, and

$$\begin{aligned} \varLambda := \lim \limits _{t \rightarrow \infty } \left[ ~\frac{ \int _{\varOmega } n_t(x) \, dx }{\int _{\varOmega } n_0(x) \, dx}\right] ^{1/t}= \lim \limits _{t \rightarrow \infty } \left[ ~ \int _{\varOmega } n_t(x) \, dx \right] ^{1/t} =r, \end{aligned}$$

independent of the initial function $n_0\in C_+(\varOmega )\backslash \{0\}$. This completes the proof of Theorem 2.

### A.5 Explicit solution for random asymmetric exponential kernels

Here we present the details for the dispersal success approximations (52) when the kernels $K_t$ are random advective kernels of the form (5), i.e.,

70 $$\begin{aligned} K_t(x) = {\left\{ \begin{array}{ll} A_t \, e^{a_{t} x} &{}\quad \text { if } x < 0 \\ A_t \, e^{b_{t} x} &{}\quad \text { if } x \ge 0, \end{array}\right. } \end{aligned}$$

where the rate constants are defined by:

$$\begin{aligned} a_{t} = \frac{v}{2D} + \sqrt{\frac{v^2}{4D^2} + \frac{\beta }{D}}, \quad b_{t} = \frac{v}{2D} - \sqrt{\frac{v^2}{4D^2} + \frac{\beta }{D}}, \quad \text {and} \quad A_t = \frac{a_{t} b_{t}}{b_{t} - a_{t}} \end{aligned}$$

where $v$ is the advection velocity, $\beta$ is the settling rate , and $D$ is the diffusion coefficient for the kernel $K_t$. The key step is the following lemma which can be shown by a direct integration:

#### **Lemma 1**

Let $\varOmega = (-L/2,L/2)$ and suppose $K_t(x-y)$ is an asymmetric advective kernel of the form (70).

If $$\gamma \notin \{a_{t},b_t\}$$, then

71 $$\begin{aligned} \int _{\varOmega } K_t(x-y) \, e^{\gamma y} \, dy = c_{0,t}(\gamma ) e^{\gamma x} + c_{a,t}(\gamma ) \, e^{a_{t} x} + c_{b,t}(\gamma ) \, e^{b_{t} x} \end{aligned}$$

where

72 $$\begin{aligned} c_{0,t}(\gamma )&= \frac{a_{t} \, b_{t}}{(\gamma - a_{t})(\gamma - b_{t})} \end{aligned}$$

73 $$\begin{aligned} c_{a,t}(\gamma )&= \frac{a_{t} \, b_{t} }{(b_{t}-a_{t})(\gamma - a_{t})} \, e^{\frac{L}{2} (\gamma - a_{t})} \end{aligned}$$

74 $$\begin{aligned} c_{b,t}(\gamma )&= \frac{-a_{t} \, b_{t} }{(b_{t}-a_{t})( \gamma -b_{t})} \, e^{-\frac{L}{2} (\gamma - b_{t})} \end{aligned}$$

Now consider the model

$$\begin{aligned} n_{t+1}(x) = R_{t} \int _{\varOmega } K_t(x-y) \, n_{t} (y) \, dy \end{aligned}$$

where $K_t$ is an asymmetric advective kernel (70). If $n_0(x) = 1$, then from Lemma 1 with $\gamma = 0$ we have

75 $$\begin{aligned} n_1(x)&= R_1 \int _{\varOmega } K_1(x-y) \, n_0(y) \, dy \end{aligned}$$

76 $$\begin{aligned}&= R_1 (c_{0,1}(0) + c_{a,1}(0) e^{a_{1} x} + c_{b,1}(0) e^{b_{1} x} ) \end{aligned}$$

where $c_{r,1}(0)$ are defined by (72)–(74). Continuing, we have

$$\begin{aligned} n_2(x)&= R_2 \int _{\varOmega } K_2(x-y) \, n_1(y) \, dy \\&= R_1 R_2 \int _{\varOmega } K_2(x-y) \, (c_{0,1}(0) + c_{a,1}(0) e^{a_{1} x} + c_{b,1}(0) e^{b_{1} x} ). \end{aligned}$$

If the rate constants $a_2,b_2 \notin \{a_1,b_1\}$ then we can apply Lemma 1 again to conclude

$$\begin{aligned} n_2(x)&= R_1 R_2 \int _{\varOmega } K_2(x-y) \, (c_{0,1}(0) + c_{a,1}(0) e^{a_{1} x} + c_{b,1}(0) e^{b_{1} x} ) \\&= R_1 R_2 \left( {\rho }_{0,2} + {\rho }_{a_1,2} e^{a_{1} x} + {\rho }_{b_1,2} e^{b_{1} x} + {\rho }_{a_2,2} e^{a_{2} x} + {\rho }_{b_2,2} e^{b_{2} x} \right) \end{aligned}$$

where

$$\begin{aligned} {\rho }_{0,2}&= c_{0,1}(0) c_{0,2}(0) \\ {\rho }_{a_1,2}&= c_{a,1}(0) c_{0,2}(a_1) \\ {\rho }_{b_1,2}&= c_{b,1}(0) c_{0,2}(b_1) \\ {\rho }_{a_2,2}&= c_{0,1}(0) c_{a,2}(0) + c_{a,1}(0) c_{a,2}(a_1) + c_{b,1}(0) c_{a,2}(b_1) \\ {\rho }_{b_2,2}&= c_{0,1}(0) c_{b,2}(0) + c_{a,1}(0) c_{b,2}(a_1) + c_{b,1}(0) c_{b,2}(b_1) \end{aligned}$$

and the constants $c_{r,j}(\theta )$ are defined by (72)–(74). We can continue in this manner to obtain $n_t(x)$ for all $t$, as long as Lemma 1 applies, which will be true provided at each step the rate constants $a_t,b_t \notin \{a_1,b_1,a_2,b_2,\dots ,a_{t-1},b_{t-1}\}$. Assuming this, we can recursively define the coefficients ${\rho }_{r,t}$ of $e^{rx}$ in $n_t(x)$ as follows. Let

$$\begin{aligned} {\rho }_{0,1} = c_{0,1}(0) \qquad {\rho }_{a_1,1} = c_{a,1}(0) \qquad {\rho }_{b_1,1} = c_{b,1}(0). \end{aligned}$$

Then, by (76), we have

$$\begin{aligned} n_1(x) = R_1 \left( \langle {\rho }_{0,1},{\rho }_{a,1},{\rho }_{b,1}\rangle \cdot \langle 1,e^{a_1x},e^{b_1x} \rangle \right) . \end{aligned}$$

Similarly, for $t=2$ we have

$$\begin{aligned} n_2(x) = R_1 R_2 \left( \langle {\rho }_{0,2},{\rho }_{a_1,2},{\rho }_{b_1,2},{\rho }_{a_2,2},{\rho }_{b_2,2}\rangle \cdot \langle 1,e^{a_1 x},e^{b_1 x},e^{a_2 x},e^{b_2 x} \rangle \right) \end{aligned}$$

where

$$\begin{aligned} \begin{bmatrix} {\rho }_{0,2} \\ {\rho }_{a_1,2} \\ {\rho }_{b_1,2} \\ {\rho }_{a_2,2} \\ {\rho }_{b_2,2} \end{bmatrix} = \begin{bmatrix} c_{0,2}(0)&\quad 0&\quad 0 \\ 0&\quad c_{0,2}(a_1)&\quad 0 \\ 0&\quad 0&\quad c_{0,2}(b_1) \\ c_{a,2}(0)&\quad c_{a,2}(a_1)&\quad c_{a,2}(b_1) \\ c_{b,2}(0)&\quad c_{b,2}(a_1)&\quad c_{b,2}(b_1) \end{bmatrix} \begin{bmatrix} {\rho }_{0,1} \\ {\rho }_{a_1,1} \\ {\rho }_{b_1,1} \end{bmatrix}. \end{aligned}$$

Similarly,

$$\begin{aligned} n_3(x)&= R_1 R_2 R_3 (\langle {\rho }_{0,3},\mathbf{\rho }_{a_1,3}, \mathbf{\rho }_{b_1,3}, \mathbf{\rho }_{a_2,3},\mathbf{\rho }_{b_2,3},\mathbf{\rho }_{a_3,3},\mathbf{\rho }_{b_3,3} \rangle \\&\cdot \langle 1,e^{a_1 x},e^{b_1 x},e^{a_2 x},e^{b_2 x} ,e^{a_3x},e^{b_3 x} \rangle ) \end{aligned}$$

where

77 $$\begin{aligned} \begin{bmatrix} {\rho }_{0,3} \\ {\rho }_{a_1,3} \\ {\rho }_{b_1,3} \\ {\rho }_{a_2,3} \\ {\rho }_{b_2,3} \\ {\rho }_{a_3,3} \\ {\rho }_{b_3,3} \end{bmatrix}&= \begin{bmatrix} c_{0,3}(0)&\quad 0&\quad 0&\quad 0&\quad 0 \\ 0&\quad c_{0,3}(a_1)&\quad 0&\quad 0&\quad 0 \\ 0&\quad 0&\quad c_{0,3}(b_1)&\quad 0&\quad 0 \\ 0&\quad 0&\quad 0&\quad c_{0,3}(a_2)&\quad 0 \\ 0&\quad 0&\quad 0&\quad 0&\quad c_{0,3}(b_2) \\ c_{a,3}(0)&\quad c_{a,3}(a_1)&\quad c_{a,3}(b_1)&\quad c_{a,3}(a_2)&\quad c_{a,3}(b_2) \\ c_{b,3}(0)&\quad c_{b,3}(a_1)&\quad c_{b,3}(b_1)&\quad c_{b,3}(a_2)&\quad c_{b,3}(b_2) \end{bmatrix} \begin{bmatrix} {\rho }_{0,2} \\ {\rho }_{a_1,2} \\ {\rho }_{b_1,2} \\ {\rho }_{a_2,2} \\ {\rho }_{b_2,2} \end{bmatrix}\nonumber \\ \end{aligned}$$

In general, $n_t(x)$ has the form

78 $$\begin{aligned} n_t(x) = {\fancyscript{R}}_t \, \left( {\rho }_{0,t} + \sum _{j=1}^{t} \left( {\rho }_{a_j,t} \, e^{a_j x} + {\rho }_{b_j,t} \, e^{b_j x} \right) \right) \end{aligned}$$

where ${\fancyscript{R}}_t =R_1 R_2 \cdots R_t$ and $\varvec{\mathrm {P}}_t = \langle {\rho }_{0,t},{\rho }_{a_1,t},{\rho }_{b_1,t},\ldots ,{\rho }_{a_t,t},{\rho }_{b_t,t} \rangle$ is defined recursively by

79 $$\begin{aligned} \varvec{\mathrm {P}}_t = \begin{bmatrix} c_{0,t}(0)&\quad 0&\quad&\quad \cdots&\quad&\quad 0 \\ 0&\quad c_{0,t}(a_1)&\quad&\quad&\quad&\\&\quad&\quad c_{0,t}(b_1)&\quad&\quad&\quad \vdots \\ \vdots&\quad&\quad&\quad \ddots&\quad&\\&\quad&\quad&\quad&\quad c_{0,t}(a_2)&\quad 0 \\ 0&\quad&\quad \cdots&\quad&\quad 0&\quad c_{0,t}(b_2) \\ c_{a,t}(0)&\quad c_{a,t}(a_1)&\quad c_{a,t}(b_1)&\quad \cdots&\quad c_{a,t}(a_{t-1})&\quad c_{a,t}(b_{t-1}) \\ c_{b,t}(0)&\quad c_{b,t}(a_1)&\quad c_{b,t}(b_1)&\quad \cdots&\quad c_{b,t}(a_{t-1})&\quad c_{b,t}(b_{t-1}) \end{bmatrix} \varvec{\mathrm {P}}_{t-1} \end{aligned}$$

By integrating (78) from $-L/2$ to $L/2$ we obtain the exact form of $\varLambda _t$:

80 $$\begin{aligned} \varLambda _t = \fancyscript{R}_t^{1/t} \left( L \, {\rho }_{0,t} + 2 \sum _{j=1}^{t} \left( \frac{{\rho }_{a_j,t}}{a_j} \sinh \frac{ a_j L}{2} + \frac{{\rho }_{b_j,t}}{b_j} \sinh \frac{ b_j L}{2} \right) \right) ^{1/t}. \end{aligned}$$
